# Mechanical Manipulation of Quantum Interference in Single‐Molecule Junctions

**DOI:** 10.1002/smll.202308865

**Published:** 2024-01-14

**Authors:** Amit Sil, Munirah Alsaqer, Chiara E. Spano, Adam Larbi, Simon J. Higgins, Craig M. Robertson, Mariagrazia Graziano, Sara Sangtarash, Richard J. Nichols, Hatef Sadeghi, Andrea Vezzoli

**Affiliations:** ^1^ Department of Chemistry University of Liverpool Crown Street Liverpool L69 7ZD UK; ^2^ Device Modelling Group School of Engineering University of Warwick Coventry CV4 7AL UK; ^3^ Department of Electronics and Telecommunications Politecnico di Torino Corso Duca degli Abruzzi Torino 10129 Italy; ^4^ Department of Applied Science and Technology Politecnico di Torino Corso Duca degli Abruzzi Torino 10129 Italy

**Keywords:** 1, 1’‐dinaphthyl, mechanical switch, mechanoresistivity, molecular electronics

## Abstract

Mechanosensitive molecular junctions, where conductance is sensitive to an applied stress such as force or displacement, are a class of nanoelectromechanical systems unique for their ability to exploit quantum mechanical phenomena. Most studies so far relied on reconfiguration of the molecule‐electrode interface to impart mechanosensitivity, but this approach is limited and, generally, poorly reproducible. Alternatively, devices that exploit conformational flexibility of molecular wires have been recently proposed. The mechanosensitive properties of molecular wires containing the 1,1’‐dinaphthyl moiety are presented here. Rotation along the chemical bond between the two naphthyl units is possible, giving rise to two conformers (*transoid* and *cisoid*) that have distinctive transport properties. When assembled as single‐molecule junctions, it is possible to mechanically trigger the *transoid* to *cisoid* transition, resulting in an exquisitely sensitive mechanical switch with high switching ratio (> 10^2^). Theoretical modeling shows that charge reconfiguration upon *transoid* to *cisoid* transition is responsible for the observed behavior, with generation and subsequent lifting of quantum interference features. These findings expand the experimental toolbox of molecular electronics with a novel chemical structure with outstanding electromechanical properties, further demonstrating the importance of subtle changes in charge delocalization on the transport properties of single‐molecule devices.

## Graphical abstract

1,1’‐dinaphthyl is an exceptionally promising molecular building block for mechanically‐switchable single‐molecule junctions. Quantum interference phenomena arising from internal charge reorganisation underpin the switching mechanism and grants enhanced sensitivity to the applied electrode displacement.

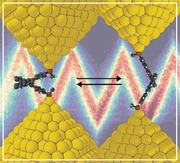

## 1 Introduction

Charge transport efficiency in single‐molecule junctions is dictated by the frontier molecular orbitals and the resulting charge distribution, which form transport eigenchannels facilitating electron tunneling from the source to the drain electrode. Important factors are i) their energy with respect to the Fermi level of the electrodes *E_F_* (source of tunneling charge carriers), and ii) their distribution in space. Good overlap between *E_F_* and the energy of the molecular orbitals yields efficient transport, while the presence of nodes in the spatial distribution can introduce quantum interference features in the transmission profile that suppress charge transport.^[^ ^1^, ^2^, ^3^ ^]^ Given the importance of molecular transport orbitals, finding ways to achieve their manipulation would grant a fine level of control on the conductance of a molecular junction. Changes in the energy and the charge distribution of molecular orbitals can be achieved, for instance, by employing ex situ synthetic processes^[^ ^4^, ^5^, ^6^, ^7^, ^8^ ^]^ or by performing in situ photochemical^[^ ^9^, ^10^, ^11^, ^12^ ^]^ or redox^[^ ^13^, ^14^, ^15^, ^16^ ^]^ reactions. In the former case, chemical synthesis is used to modify the structure of the molecular wire to induce changes in the molecular orbitals by introducing electron‐withdrawing/electron‐donating groups that shift the overall charge distribution. In the case of in situ redox chemistry, this is achieved by chemically or electrochemically oxidising or reducing the molecular wire to its corresponding *n* ± 1 charge state, where the molecular orbitals have completely different energies and spatial distribution. These two methods, while highly effective, have the main drawback of not allowing either reversibility in the case of ex situ substitution chemistry, or a fine level of gradation for in situ redox switching.

Mechanical manipulation of the molecular junction as a way of changing its charge transport efficiency is an enticing alternative.^[^ ^17^ ^]^ Through mechanosensitive phenomena (dependence of charge transport efficiency on applied force or mechanical displacement) a fine level of control on junction conductance has been achieved, for instance, by exploiting interfacial effects at the pyridyl,^[^ ^18^, ^19^ ^]^ thienyl^[^ ^20^ ^]^ or carbodithioate^[^ ^21^ ^]^ electrode contact or in carotenoid molecular wires,^[^ ^22^ ^]^ by using molecules with multiple metallophilic groups spaced along the conductive backbone,^[^ ^23^, ^24^, ^25^, ^26^ ^]^ or by employing flexible molecules that can fold/unfold (synthetic foldamers),^[^ ^27^, ^28^, ^29^ ^]^ rotate,^[^ ^30^ ^]^ or shear^[^ ^31^, ^32^ ^]^ along an axis. Of all these methods, only the use of flexible molecules provides real mechanical manipulation of the transport orbitals. Destructive quantum interference arising from orbital symmetry results has been demonstrated to impart mechanosensitivity to ferrocenyl^[^ ^30^ ^]^ and [2,2]paracyclophanyl^[^ ^32^ ^]^ molecular wires (in both cases the quantum interference feature is lifted upon junction compression), while through‐space interactions between fragments of the molecular wire brought into close proximity by junction compression underpin the mechanosensitive behaviour of synthetic foldamers.^[^ ^27^, ^28^ ^]^

**Figure 1:**
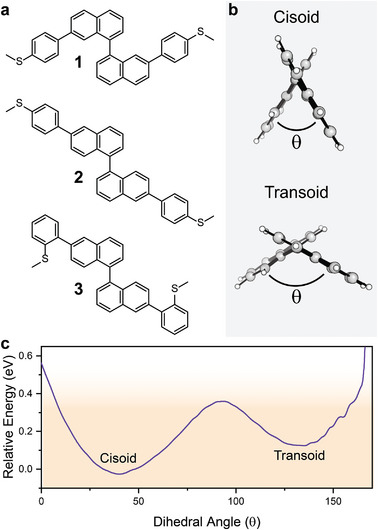
a) Structure of the molecular wires used in this study. b) Axial view of the 1,1’‐binapthyl system in its *cisoid* and *transoid* configuration, viewed through the napthyl‐napthyl bond axis. c) Energy profile (molecular mechanics, MM2 force field) for the rotation around the napthyl‐napthyl bond of **1**. The energy range accessible through mechanical compression in single‐molecule junctions assembled with 4‐thioanisolyl termini is shown as shaded orange area.

In this contribution, we show that the 1,1’‐dinaphthyl moiety (derivatives of interest in **Figure** **1**a) is a mechanosensitive building block with excellent repeatability/cyclability and very large modulation range. In this case, mechanical manipulation of the naphthyl‐naphthyl dihedral angle changes the overall charge distribution in the system and introduces quantum interference phenomena, in the form of antiresonances in the transmission profile. The energy of the antiresonances can be mechanically introduced, controlled, and lifted, resulting in exquisite control exerted over charge transport efficiency.

## 2 Results and Discussion

The 1,1’‐dinaphthyl moiety has rich substitution chemistry, and we explored it by synthesizing species with 4‐thioanisolyl termini in the positions 7,7’ (**1** in Figure 1a) and 6,6’ (**2** and **3** in Figure 1a). The synthetic procedures for compounds **1**–**3** (as racemic mixtures, spontaneously equilibrating in solution^[^ ^33^ ^]^ during synthesis) are reported in the Supporting Information, along with their characterization. Molecular modeling on **1** demonstrates the promise of the 1,1’‐binapthyl system as a mechanosensitive molecular switch. The energy profile for changes in the dihedral angle between the two naphthyl units shows two minima, corresponding to the *cisoid* (dihedral angle θ ≅ 40 ^○^) and *transoid* (θ ≅ 130°) configurations (Figure 1b), a phenomenon reflected in the reported crystallographic polymorphism of 1,1’‐dinaphthyl. The two structures (*cisoid* and *transoid*) have indeed been isolated and characterized by single‐crystal X‐ray diffraction.^[^ ^34^ ^]^ The interconversion barrier between the two configurations is shallow and readily accessible within the energetics of the Au‐SR coordination interaction (0.35–0.7 eV),^[^ ^27^, ^35^, ^36^, ^37^ ^]^ pointing toward the opportunity to mechanically force the interconversion in 1,1’‐binapthyl derivatives when assembled as a single‐molecule junction using widely used methyl sulfide anchors (Figure 1c).^[^ ^38^, ^39^ ^]^

Having established through preliminary modeling that the 1,1’‐binapthyl system has great potential to behave as a mechanosensitive switch, we started our experimental investigation by performing scanning tunneling microscopy – break junction experiments (STMBJ)^[^ ^40^ ^]^ on the three molecular wires. These experiments repeatedly fabricate and rupture single‐molecule junctions by driving a sharp Au tip into contact with a Au substrate, and then withdrawing it at constant speed (10 *nm* *s* ^−1^ in this study). During each cycle, a Au–Au contact is fabricated, thinned down to an atomic contact, and ruptured. Upon rupture of the metallic contact, molecules with appropriate aurophilic termini (in this case, the 4‐thioanisolyl groups of **1–3**) can self‐assemble in the nanogap between the two electrodes. The tip is further withdrawn to stretch the junction to its maximum length, until it eventually breaks off. The process is performed in a liquid environment with the target molecule present in 1 mm concentration, and under a constant DC bias. The junction conductance is continuously monitored through a transimpedance amplifier as $G\;=\frac{I}{V}\;$, as a function of the quantum of conductance $G_{0}=\frac{2e^{2}}{h}\;=\;7.748\;\times 10^{-5}\;S$. The process is repeated thousands of times to acquire meaningful statistics and data is compiled in histograms and 2D maps to show the distribution of conductance values. Details on the instrumentation used in this study and our data acquisition and analysis protocols are briefly described in the SI, while more exhaustive accounts can be found in our previous publications on the subject.^[^ ^20^, ^27^ ^]^

**Figure 2:**
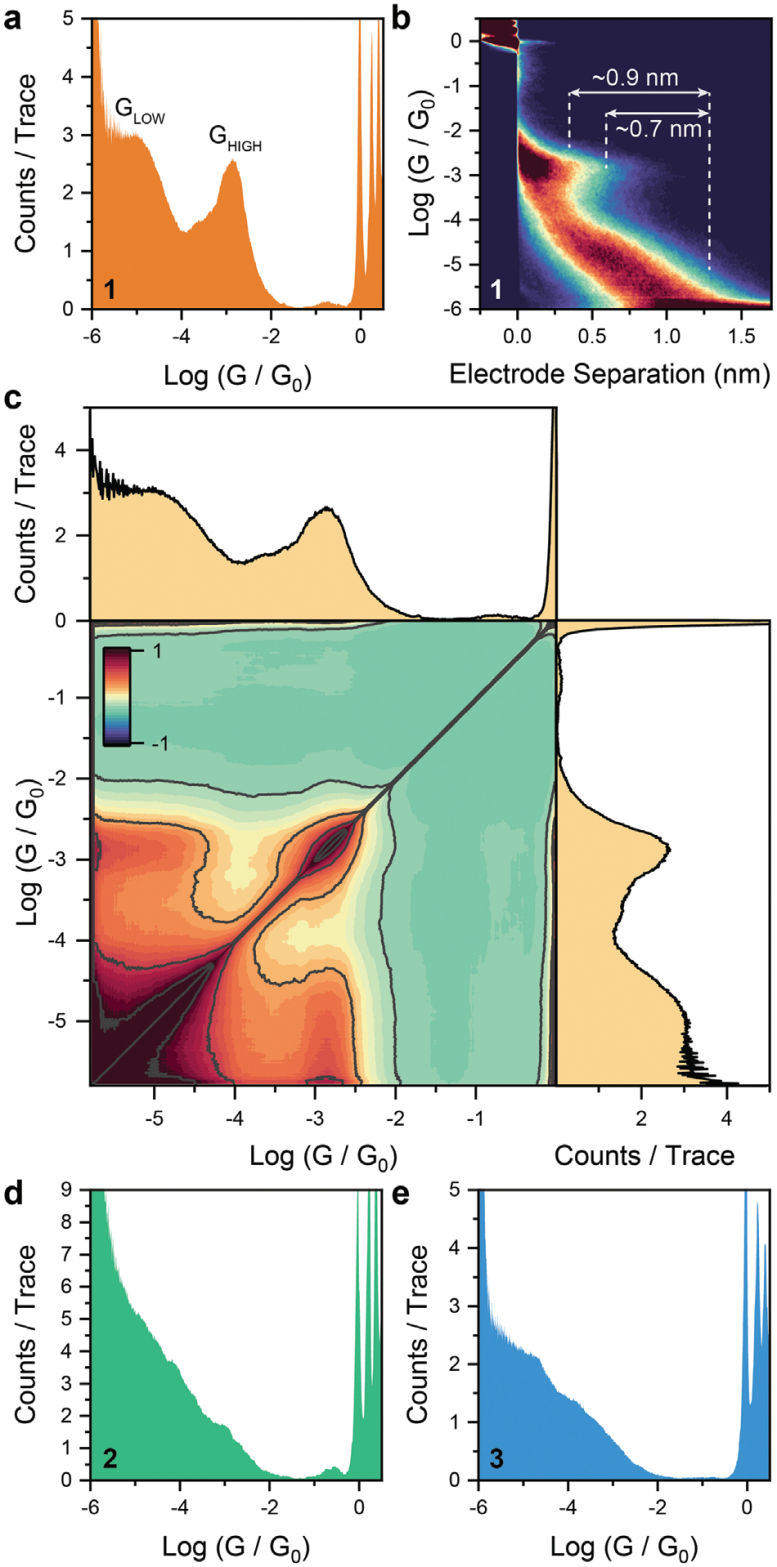
STMBJ experiments on **1**–**3**. a) Conductance histogram, b) 2D heatmap, and c) 2D correlogram for **1**. 6025 traces, 750 mV bias. d) Conductance histogram for **2**. 3581 traces, 1 V bias. e) Conductance histogram for **3**. 2950 traces, 1 V bias. All data have been acquired in 1 mm solution of the target wire in mesitylene. Plots obtained with 100 bins per conductance decade and 100 bins per nm.

The results of STMBJ experiments on **1–3** are shown in **Figure** **2**. Compound **1** shows multiple conductance peaks in the histogram and in the associated heatmap. Fabricated junctions display relatively high conductance of ≈10^−2.7^ *G*_0_ at low electrode separation. As the junction is stretched conductance falls to lower values until eventually setting to ≈10^−4.9^ *G*_0_. Such low values of conductance in the fully extended configuration are not surprising, as a fully coplanar configuration of **1** is energetically inaccessible due to steric hindrance (see Figure 1c). Therefore, while there is indeed a continuous conjugation pathway in **1**, the degree of electronic communication will be restricted. Analysis of the 2D conductance/electrode separation heatmaps shows a difference of 0.9–0.7 nm between the low and high conductance features (respectively, ≈0.5 and ≈1.3 nm, not accounting for electrode snapback). We performed further analysis to verify that the high and low conductance features in the STMBJ histogram are correlated –, *i.e*., their presence in single STMBJ traces is not mutually exclusive – by using a method originally developed for atomic point contacts.^[^ ^41^ ^]^ The correlogram in Figure 2c shows strong, positive off‐diagonal correlation in the region corresponding to the two conductance peaks in the histograms (also shown for clarity), thus strongly suggesting a mechanism of junction evolution where the molecule sits in a compressed conformation (acute dihedral angle θ) at low electrode separation, relaxing to its extended conformation (obtuse dihedral angle θ) as the electrodes are withdrawn. On the other hand, **2** and **3** did not show any prominent feature in the conductance histogram (Figure 2d,e). Analysis of the single conductance traces and of the heatmaps suggest that the compounds are poorly conductive, and their most probable conductance lies below the sensitivity of the instrumentation. Indeed, simple analytical models^[^ ^42^ ^]^ predict the presence of destructive quantum interference features arising from the 1,6‐connectivity of the napthyl moiety,^[^ ^43^ ^]^ which are known to strongly suppress charge‐transport. Theoretical modeling also confirms lower transmission coefficients for **2** and **3**, and further details can be found in the SI.

We therefore focussed our attention on **1** and performed piezo‐modulation experiments^[^ ^17^ ^]^ to further characterize the mechanosensitive behavior of its single‐molecule junctions. In these experiments, a custom signal is imposed on the piezoelectric transducer responsible for moving the STM tip in the *z*‐axis. The signal is a sum of a step function and a continuous modulation. The step function generates nanoelectrode gaps of size commensurate to molecular length and stabilizes them for 100 ms, while the modulation continuously compresses and then stretches the junction by a few Å. In these experiments, data is recorded as a function of time (rather than as a function of electrode displacement) and, again, thousands of individual traces are recorded for each target molecule and analyzed statistically as 2D heatmaps. Details of the instrumentation used in these experiments and of the data analysis protocol are described in our previous publications on the subject.^[^ ^20^, ^27^ ^]^

**Figure 3:**
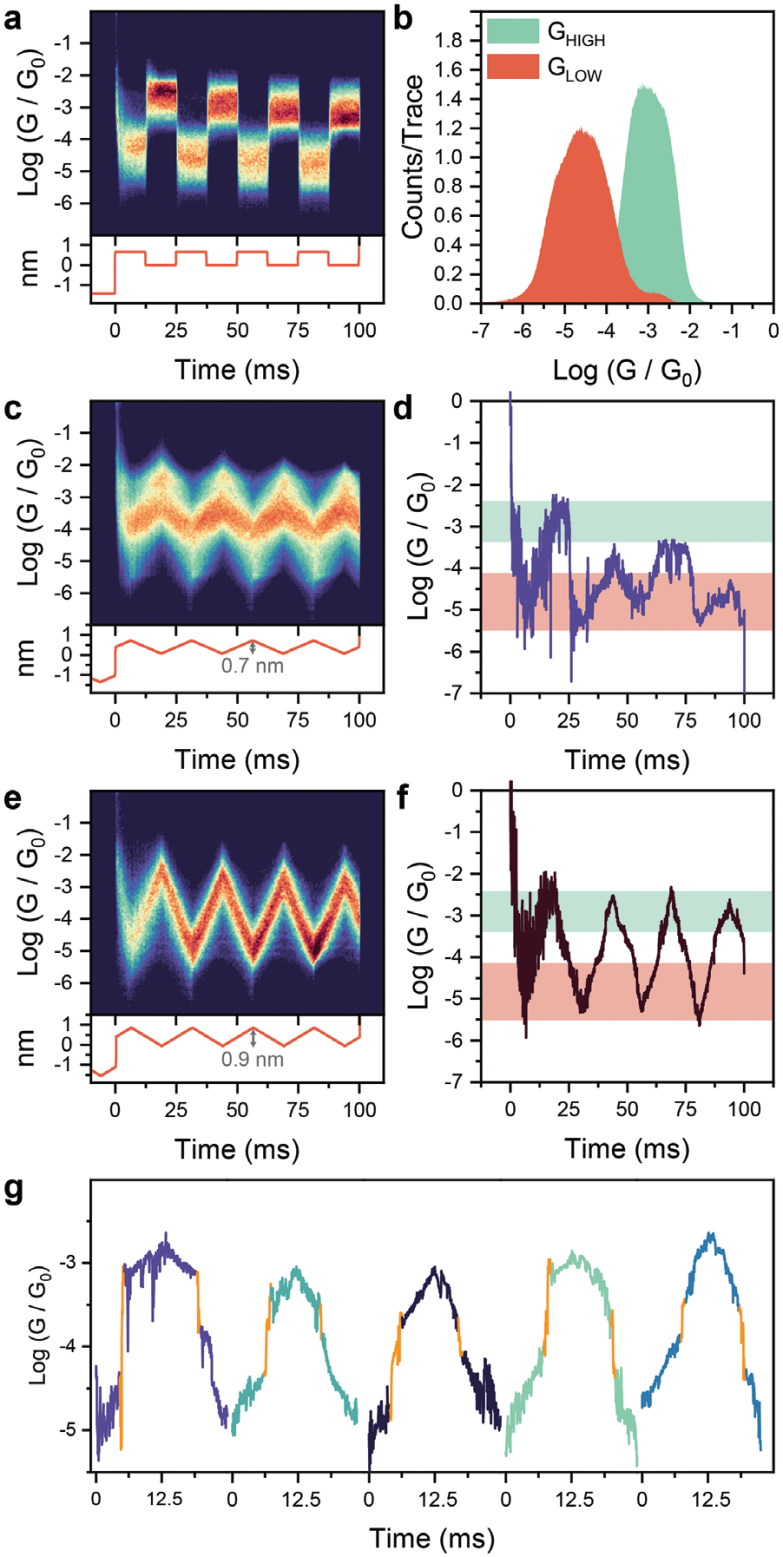
Mechanosensitive behavior of **1**. a) Conductance heatmap of **1** (top) under 40 Hz square‐wave piezo‐modulation with a 7 Å amplitude (2375 traces), and b) resulting 1D conductance histogram. c) Conductance heatmap of **1** under 40 Hz triangular piezo‐modulation with a 7 Å amplitude (3674 traces) and d) example trace. e) Conductance heatmap of **1** under 40 Hz triangular piezo‐modulation with 9 Å amplitude (2019 traces) and f) example trace. g) Five representative 9 Å modulation traces showing “hard switching” between two states, highlighted in orange. Signal imposed to piezoelectric transducer reproduced below the respective heatmap, in (a), (c), and (e). All data acquired at 0.75 V bias, in a 1 mm mesitylene solution of **1**. All heatmaps compiled with 100 bins per conductance decade and 2000 bins per second.

Under square‐wave modulation conditions (**Figure** **3**a), **1** was reliably cycled between a low and a high conductance regime, with values similar to those obtained in the regular STMBJ experiments (Figure 3b) and a switching ratio *G_HIGH_* / *G_LOW_* ≅130. To better characterise the mechanosensitive behavior of **1**, we performed triangular‐wave modulation, designed to verify the presence of either abrupt changes in conductance (“hard” switching) or more “soft” processes of continuous modulation of charge transport efficiency in phase with the piezo signal. We tested triangular waves with various amplitudes. Triangular modulation with an amplitude of 7 Å failed to reliably cycle the junction between the high and low conductance state, resulting in unclear 2D heatmaps, with most traces showing only small modulations and only few cycles attaining the high‐conductance feature observed in the square‐wave modulation (see example modulation trace in Figure 3d). This unclear modulation can be attributed to small changes in the molecule‐electrode interface as the junction is compressed,^[^ ^44^ ^]^ as the modulation amplitude is not enough to trigger the *cisoid* ⇌ *transoid* switch. We therefore performed a measurement with an amplitude of 9 Å that delivered much better results, consistent now with a fully reversible *cisoid* ⇌ *transoid* switch, and associated with a well‐defined statistical heatmap (Figure 3e, with example trace in Figure 3f).

Interestingly, a “hard” switch between sections of the trace with different slope (i.e., different mechanical sensitivity) can be seen in > 90% of the individual modulation events (some examples in Figure 3g). The position and magnitude of the switch are not consistent across the dataset, which we attribute to the fact we have no control on the initial geometry of the junction – each junction assembles in a unique configuration and has unique switching properties. Therefore, while the statistical average presented in Figure 3e may suggest a “soft” switching mechanism, this is only due to the stochastic position of the switching event, and the single modulation traces demonstrate instead evidence of “hard” switching, arising from the presence of an interconversion energy barrier (Figure 1c).

**Figure 4:**
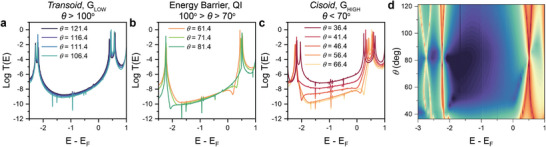
DFT transport calculations. Transmission coefficient for junctions of **1** with a naphthyl‐naphthyl dihedral angle a) θ > 100°, b) 100° > θ > 70°, and c) θ < 70°. d) Full map of the transmission coefficient as a function of the dihedral angle θ. Color scale goes from *T*(*E*) = 10^−10^ (dark purple) to *T*(*E*) = 1 (red).

To better understand the phenomena in play in **1** responsible for its mechanosensitivity, we performed Density Functional Theory (DFT) modelling using SIESTA^[^ ^45^ ^]^ and the GOLLUM code for transport calculations.^[^ ^46^, ^47^ ^]^ We calculated the zero‐bias transmission coefficient for **1** sandwiched between two Au leads as a function of the dihedral angle between the two napthyl units. In the *transoid* configuration, at θ > 100° (**Figure** **4**a), changes in the dihedral angle have very little effect on the transmission coefficient. As the dihedral angle is reduced to the range of the interconversion energy barrier (100° > θ > 70°, Figure 4b), quantum interference features start to appear near the LUMO and/or near the HOMO, suppressing transport and keeping the transmission coefficient low. At θ < 70°, instead, reduction of the dihedral angle results in significant increase in mid‐bandgap transmission (Figure 4c), arising from the lifting of the quantum interference feature and aided by the formation of π − π transport channels between the thioanisolyl rings.^[^ ^27^ ^]^ Data has been compiled in a 2D heatmap to better appreciate the generation, energetic shift, and changes in quantum interference pattern (dark purple areas in Figure 4d) as a function of the naphthyl‐naphthyl dihedral angle.

As discussed in the introduction, rotation around the naphthyl‐naphthyl bond in **1** is not unhindered like in ferrocene derivatives^[^ ^30^ ^]^ and therefore not all structures investigated by DFT are experimentally realistic. As the junction is continuously compressed it is unlikely it adopts any of the high‐energy configurations with dihedral angle 100° > θ > 70°, and a “hard”, abrupt switch from a *transoid* to a *cisoid* configuration is therefore key to achieving mechanosensitivity. When the molecule is assembled in the *transoid* configuration (local thermodynamic minimum, Figure 1c), mechanical compression can change the dihedral angle but only with relatively small effects on conductance, until further compression causes a sudden switch to a more stable *cisoid* configuration (absolute thermodynamic minimum, Figure 1c), effectively bypassing the high‐energy structures displaying dominant quantum interference (transmission profiles in Figure 4b). The *cisoid* configuration still suffers quantum interference that suppress transport (see for instance the curves in Figure 4c at ≈ 56° and ≈ 46°) but the interference is eventually lifted at sufficiently small naphthyl‐napthyl dihedral angles due to the formation of new conductance pathways through the overlap between π‐orbitals of the two naphtyls.^[^ ^27^ ^]^

**Figure 5:**
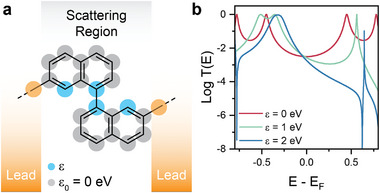
Tight‐binding model of the 1,1’‐dinaphthyl system. a) Structure of the model, with one orbital per atom connected weakly to 1D leads from the connection points shown by the dashed lines. The on‐site energies for the grey sites ε_0_ are 0 eV, while the blue sites energy ε is increased from 0 to 2 eV to simulate the change in dihedral angle θ. Coupling integrals between all sites are set at ‐1 eV. (b) Resulting transmission curves for ε = 0, 1, and 2 eV showing the development of quantum interference phenomena.

We then turned our attention to understanding the origin of the quantum interference features. Analysis of the charge distribution in **1** shows that the Mulliken charge on the atoms highlighted in blue in **Figure** **5**a changes significantly when the angle between the two naphthyl units is modified. We used this information to construct a tight‐binding model of the 1,1’‐dinaphthyl core of **1**, with one orbital per atom connected to 1D electrodes. As can be observed in Figure 5b, simply by modifying the on‐site potential energies of these atoms from 0 to 2 eV we were able to reproduce the behaviour observed in the DFT calculations, with a shift from a regular mid‐bandgap behavior with ε  =  0 eV to destructive interference with ε  =  2 eV. It is worth noting that, as discussed in other studies,^[^ ^48^, ^49^ ^]^ the electronic coupling between the two naphtyl units are also modified by θ, leading to the changes in the amplitude of transmission functions. However, this does not change the quantum interference pattern (see Figure S31, Supporting Information). We, therefore, attribute the changes in quantum interference pattern as a function of θ to the redistribution of charge over the core of **1**.

To confirm these findings, we analyzed the wavefunction of **1** in the *transoid* and *cisoid* configuration. As shown in Figure S33 (Supporting Information), the frontier molecular orbitals of **1** change with the compression of the junction due to the rotation around the naphthyl‐naphthyl bond. The sign of the product of the LUMO wavefunction at the connection points to the electrodes (*i.e*., on the S termini) switches as **1** undergoes *transoid*‐to‐*cisoid* restructuring, while the HOMO remains unchanged. The transmission coefficient *T_ij_* (*E*) between site *i* and *j* is proportional to the Green's function *g_ij_* of a molecule, defined as $g_{ij}\left(E\right)\;=\frac{\psi_{i}^{H}\psi_{j}^{H}}{E-E_{H}}\;+\frac{\psi_{i}^{L}\psi_{j}^{L}}{E-E_{L}}$. In this expression, $\psi_{b}^{a}$ represents the wavefunction at site *b* for state *a*, *E_a_* is the energy level associated with this state and *a*  =  *H*,  *L* denote HOMO and LUMO, respectively. *g_ij_* will vanish at a certain energy if the sign of product $\psi_{i}^{H}\psi_{j}^{H}$ is the same as $\psi_{i}^{L}\psi_{j}^{L}$. Therefore, the QI pattern changes from constructive in the *transoid* (relaxed) configuration to destructive in the *cisoid* (compressed) state. This is also reflected in the transmission coefficient calculations shown in Figure 4 where an antiresonance in the *T*(*E*) of **1** appears upon junction compression.

## 3 Conclusion

We demonstrated here that 1,1’‐dinaphthyl is a novel mechanosensitive functional group for molecular electronics applications, with large ON/OFF ratios and excellent reproducibility. The electromechanical properties arise from the force‐induced interconversion between two possible, thermodynamically accessible structures: a long, poorly‐conductive *transoid* configuration at large napthyl‐naphthyl dihedral angle θ > 100°, and a shorter, more efficient *cisoid* configuration at θ < 70°. The conductance traces show abrupt changes in conductance upon junction compression and stretching, revealing a “hard” switching mechanism, where the high‐energy structures in the 100° < θ < 70° range are effectively bypassed. Computational modelling identifies mechanically‐induced quantum interference phenomena, arising from changes in charge delocalisation, being generated and subsequently lifted during junction compression, explaining the large magnitude of the observed behaviour. With this study, we expanded the range of structures that can be used to impart mechanical sensitivity to molecular wires, thereby providing novel and synthetically accessible design principles for nanoelectromechanical systems based on single‐molecule junctions.

## Conflict of Interest

The authors declare no conflict of interest.

## Supporting information

Supporting Information

## Data Availability

The data that support the findings of this study are openly available in the University of Liverpool Data Catalogue at https://doi.org/10.17638/datacat.liverpool.ac.uk/2259, reference number [2259].
