# Supplementary material for: Mechanical Manipulation of Quantum Interference in Single‐Molecule Junctions
Source: Small. 2024 Jan 14;20(25):2308865. doi: 10.1002/smll.202308865 (PMC11475491; doi:10.1002/smll.202308865)
Supplement: Supplementary file 1 — Supporting Information [file SMLL-20-2308865-s001.pdf]

NANO · MICRO
small

Supporting Information

for *Small*, DOI 10.1002/smll.202308865

Mechanical Manipulation of Quantum Interference in Single-Molecule Junctions

Amit Sil, Munirah Alsaqer, Chiara E. Spano, Adam Larbi, Simon J. Higgins, Craig M. Robertson, Mariagrazia Graziano, Sara Sangtarash, Richard J. Nichols, Hatef Sadeghi and Andrea Vezzoli**

Mechanical Manipulation of Quantum Interference in Single-Molecule Junctions

Amit Sil,[a,#] Munirah Alsaqer,[b,#] Chiara E. Spano,[a,c] Adam Larbi,[a] Simon J. Higgins,[a] Craig M. Robertson,[a] Mariagrazia Graziano,[d] Sara Sangtarash,[b] Richard J. Nichols,[a] Hatef Sadeghi,[b,*] Andrea Vezzoli.[a,*]

a) Department of Chemistry, University of Liverpool, Crown Street, Liverpool L69 7ZD, United Kingdom

b) Device Modelling Group, School of Engineering, University of Warwick, Coventry CV4 7AL, United Kingdom

c) Department of Electronics and Telecommunications, Politecnico di Torino, Corso Duca degli Abruzzi, 10129 Torino, Italy

d) Department of Applied Science and Technology, Politecnico di Torino, Corso Duca degli Abruzzi, 10129 Torino, Italy

#: these authors contributed equally to this work

*: corresponding authors: andrea.vezzoli@liverpool.ac.uk

hatef.sadeghi@warwick.ac.uk

Contents:

1. Synthetic Details 3

1.1. Synthesis of 1 3

Synthesis of 7,7'-dimethoxy-3,3',4,4'-tetrahydro-1,1'-binaphthalene (4) 3

Synthesis of 7,7'-dimethoxy-1,1'-binaphthalene (5) 4

Synthesis of 1,1'-binaphthalene]-7,7'-diol (6) 4

Synthesis of [1,1'-binaphthalene]-7,7'-diyl bis(trifluoromethanesulfonate) (7) 5

Synthesis of 7,7'-bis(4-(methylthio)phenyl)-1,1'-binaphthalene (1) 5

1.2. Synthesis of 2 6

Synthesis of naphthalene-1,6-diyl bis(trifluoromethanesulfonate) (8) 6

Synthesis of 6-(4-(methylthio)phenyl)naphthalen-1-yl trifluoromethanesulfonate (9) 7

Synthesis of 4,4,5,5-tetramethyl-2-(6-(4-(methylthio)phenyl)naphthalen-1-yl)-1,3,2-dioxaborolane (10) 7

Synthesis of 6,6'-bis(4-(methylthio)phenyl)-1,1'-binaphthalene (2) 8

1.3. Synthesis of 3 8

Synthesis of 6,6'-bis(2-(methylthio)phenyl)-1,1'-binaphthalene (9, AS118) 9

2. NMR Spectra 10

3. Details on STMBJ measurements 21

3.1. STMBJ Measurements 21

3.2. Piezo-Modulation STMBJ Measurements 22

3.3. Correlation Plots 23

3.4. Data and Software availability 23

4. Additional STMBJ Data 24

5. Computational Methods 25

References 28

1. Synthetic Details

Reagents were purchased from Merck, TCI or Fluorochem, depending on availability. Solvents were purchased from ThermoFisher scientific. Anhydrous solvents were obtained from a solvent purification system (Innovative Technology Puresolv) and stored over molecular sieves prior to use.

NMR spectra were recorded on a Bruker Avance III HD spectrometer. Chemical shifts δ are reported in ppm, referenced against the residual solvent and internal TMS standard. Flash chromatography was performed on Silica gel 40-63 µm (230-400 mesh, Merck). Mass spectrometry was carried out on an Agilent QTOF 7200 in EI or CI ionisation.

1.1. Synthesis of 1

The synthesis of compound **1** was performed as in the scheme below, starting from 7-methoxy-3,4-dihydronaphthalen-1-one, which was dimerised with Zn and trimethylsilyl chloride, followed by reduction with trityl fluoroborate and demethylation with boron tribromide to yield 1,1'-binapthalen-7,7'-diol **6**. The diol was converted to the ditriflate **7**, followed by Pd-catalysed Suzuki cross-coupling with 4-(methylthio)phenylboronic acid to yield **1**.

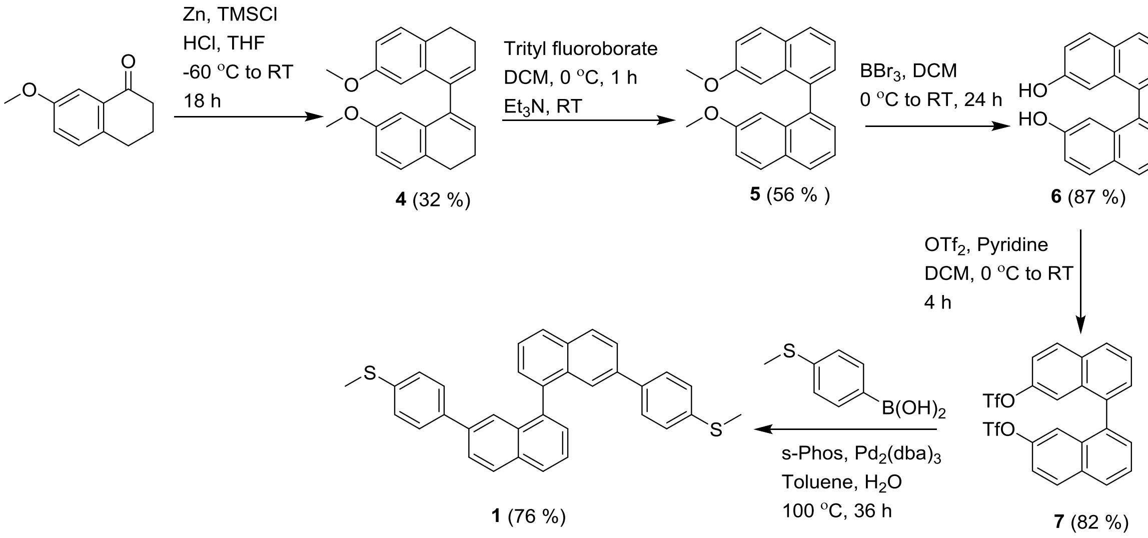

Synthesis of 7,7'-dimethoxy-3,3',4,4'-tetrahydro-1,1'-binaphthalene (4)

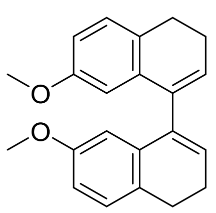

An oven dried flask equipped with a stir bar was charged with Zn (0.62 g, 9.48 mmol) and 95 mL of anhydrous THF, under an inert atmosphere. The suspension was then cooled down to -60 °C. 6-methoxy-1-tetralone (3.0 g, 17.02 mmol), TMSCl (4.06 g, 37.4 mmol) and 2.5 ml of HCl 37% were subsequently added. The solution was left under stirring at -50 °C for 30 minutes and then allowed to warm to room temperature overnight. The reaction was then quenched with 50 mL of water and diluted with 150 mL of diethyl ether. After extraction, the organic phase was

washed with brine and water, dried over anhydrous MgSO4 and filtered. All the volatilities were removed under reduced pressure. The crude product was purified by column chromatography over silica gel, eluting with a hexanes:dichloromethane mixture (80:20 *v:v*) to afford the title compound 5 as a pale brown oil. (1.65 g, 32 %). 1H NMR (500 MHz, CDCl3) δ 7.08 (d, J = 8.2 Hz, 2H), 6.65 (dd, J = 8.2, 2.6 Hz, 2H), 6.56 (d, J = 2.6 Hz, 2H), 6.10 (t, J = 4.5 Hz, 2H), 3.64 (s, 6H), 2.82 (d, J = 7.0 Hz, 4H), 2.39 (dt, J = 12.1, 6.2 Hz, 4H).; 13C NMR (126 MHz, CDCl3) δ 158.3, 138.4, 136.0, 128.9, 128.4, 128.1, 111.9, 111.4, 55.3, 27.6, 23.9. HRMS (CI) m/z = 319.1695 [M+H]+; calculated for $[C_{22}H_{23}O_2]^+$ 319.1698.

Synthesis of 7,7'-dimethoxy-1,1'-binaphthalene (5)

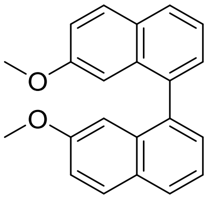

To a 250 ml Schlenk flask with a stir bar was added compound **4** (1.65 g, 5.18 mmol) and dry dichloromethane (60 ml) under inert atmosphere. The solution was cool down to 0 °C and then trityl fluoroborate (4.28 g, 12.95 ml) was added by small portion and the reaction mixture was stirred for an hour at 0 °C. Then triethyl amine (6.50 ml, 46.62 mmol) was added to the solution dropwise. The resulting mixture was filtered over a pad of silica gel and washed with dichloromethane then the filtrate was concentrated under vacuum. The crude product was purified by column chromatography over silica gel with petroleum ether : dichloromethane mixture (*v:v,* 80:20) as eluent to afford the title compound **11** as a white solid. (0.92 g, 56 %). ^{1}H NMR (500 MHz, $CDCl_3$) δ 7.96 – 7.78 (m, 4H), 7.66 – 7.41 (m, 4H), 7.18 (dd, *J* = 8.9, 2.3 Hz, 2H), 6.79 (dd, *J* = 7.3, 2.5 Hz, 2H), 3.56 (s, 6H). ^{13}C NMR (126 MHz, $CDCl_3$) δ 157.6, 137.4, 133.7, 129.7, 129.2, 128.4, 127.6, 123.2, 118.4, 104.9, 55.1. HRMS (CI) *m/z* = 315.1379 $[M+H]^+$; calculated for $[C_{22}H_{19}O_2]^+$ 315.1385.

Synthesis of 1,1'-binaphthalene]-7,7'-diol (6)

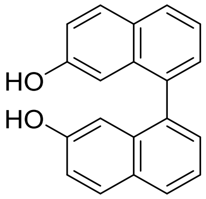

A dry 100 ml Schlenk flask compound **5** (1.16 g, 3.71 mmol) was dissolved in 45 ml anhydrous CH_2Cl_2 in inert atmosphere and the mixture was cooled down to 0 °C. Then boron tribromide solution (15 ml, 22.27 mmol) (1.0 M in DCM) were added dropwise and the reaction mixture was stirred overnight. The reaction mixture was hydrolysed carefully at 0 °C. Then the organic phase was washed with brine, water and dried over anhydrous MgSO4. All the volatilities were removed under reduced pressure. The crude product was purified by column chromatography using

DCM as an eluent to obtain an off white solid (0.92 g, 87%); ^{1}H NMR (500 MHz, $CDCl_3$) δ 7.88 – 7.78 (m, 4H), 7.49 – 7.37 (m, 4H), 7.08 (dd, *J* = 8.8, 2.5 Hz, 2H), 6.65 (d, *J* = 2.3 Hz, 2H). ^{13}C NMR (126 MHz, $CDCl_3$) δ 153.8, 137.1, 134.1, 130.3, 129.2, 128.5, 127.8, 123.4, 117.9, 108.5. HRMS (CI) *m/z* = 287.1061 $[M+H]^+$; calculated for $[C_{20}H_{15}O_2]^+$ 287.1072.

Synthesis of [1,1'-binaphthalene]-7,7'-diyl bis(trifluoromethanesulfonate) (7)

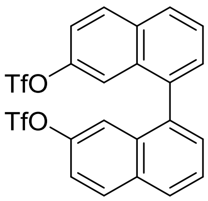

In an oven dried Schlenk flask purged with nitrogen, compound **6** (0.59 g, 2.05 mmol), anhydrous pyridine (1.0 ml, 12.33 mmol) were dissolved in 20 ml of anhydrous DCM under N_2 atmosphere. The mixture was cooled down to 0 °C and trifluoromethanesulfronic anhydride (1.0 ml, 6.16 mmol) of was added dropwise. The reaction mixture was stirred for 30 min at 0 °C and slowly warmed to room temperature and stirred for 2 hours. Then the reaction was quenched with water and the organic phase was separated and washed with brine, water. The organic extract was dried over anhydrous $MgSO_4$, filtered and all the volatilities were removed under reduced pressure. The crude product was purified by silica gel flash column using petroleum ether and CH_2Cl_2 (50:50) as an eluent to obtain a thick pale-yellow oil (0.92 g, 82%). ^{1}H NMR (500 MHz, $CDCl_3$) δ 8.06 (t, *J* = 8.9 Hz, 4H), 7.72 (dd, *J* = 8.2, 7.2 Hz, 2H), 7.60 (dd, *J* = 7.0, 0.8 Hz, 2H), 7.48 – 7.39 (m, 2H), 7.25 (d, *J* = 2.4 Hz, 2H). ^{13}C NMR (126 MHz, $CDCl_3$) δ 147.8, 137.5, 132.9, 132.9, 131.4, 129.8, 128.7, 126.9, 120.1, 117.9. ^{19}F NMR (471 MHz, $CDCl_3$) δ -72.93. HRMS (CI) *m/z* = 551.0058 $[M+H]^+$; calculated for $[C_{22}H_{13}F_6O_6S_2]^+$ 551.0046.

Synthesis of 7,7'-bis(4-(methylthio)phenyl)-1,1'-binaphthalene (1)

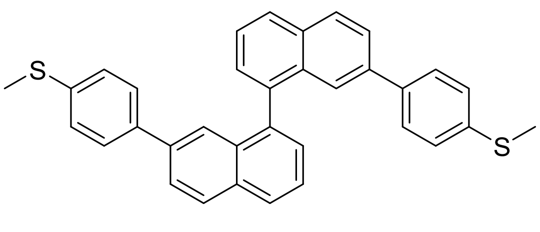

A mixture of compound **13** (450 mg, 0.81 mmol), (4-(methylthio)phenyl)boronic acid (412 mg, 2.45 mmol), K_3PO_4 (1.04 g, 4.90 mmol), and toluene/ water (10 mL/ 1 mL) was degassed by three freeze-pump-thaw cycles. $Pd_2(dba)_3$ (38 mg, 0.04 mmol) and S-Phos (34 mg, 0.08 mmol) were added under a flow of nitrogen and the resulting solution was heated to 100 °C for 36 h. The mixture was allowed to cool to room temperature and the solvents were removed under vacuum. The organic part was extracted in dichloromethane and the organic extract was washed with brine, dried over

anhydrous magnesium sulfate, filtered and the solvent was removed. The crude material was purified by flash column chromatography over silica gel with hexanes: ethyl acetate mixture (*v:v,* 95:5) as eluent, followed by recrystallisation from $CHCl_3$/pentane to afford the title compound **14** as white solid (309 mg, 76 %). ^{1}H NMR (500 MHz, $CDCl_3$) δ 8.01 (d, *J* = 8.5 Hz, 2H), 7.97 (d, *J* = 8.1 Hz, 2H), 7.72 (dd, *J* = 8.5, 1.8 Hz, 2H), 7.65 (s, 2H), 7.63 – 7.57 (m, 2H), 7.54 (dd, *J* = 6.9, 1.0 Hz, 2H), 7.35 – 7.30 (m, 4H), 7.19 (dd, *J* = 8.6, 1.9 Hz, 4H), 2.44 (s, 6H). ^{13}C NMR (126 MHz, $CDCl_3$) δ 138.7, 138.3, 138.2, 137.8, 133.3, 133.0, 129.1, 128.7, 127.9, 127.9, 127.1, 125.7, 125.6, 124.3, 16.1. HRMS (CI) *m/z* = 499.1525 $[M+H]^+$; calculated for $[C_{34}H_{24}S_2]^+$ 499.1554.

1.2. Synthesis of 2

Compound **2** was synthesised starting from naphthalen-1,6-diol, by triflation of the diol, followed by Pd-catalyised Suzuki cross-coupling with 4-(methylthio)phenylboronic acid, to give the intermediate 6-(4-(methylthio)phenyl)naphthalen-1-yl trifluoromethanesulfonate **9**. A portion of this was subjected to Miyaura borylation to yield the boronic ester **10**, which was coupled to **9** under Suzuki conditions to give **2**.

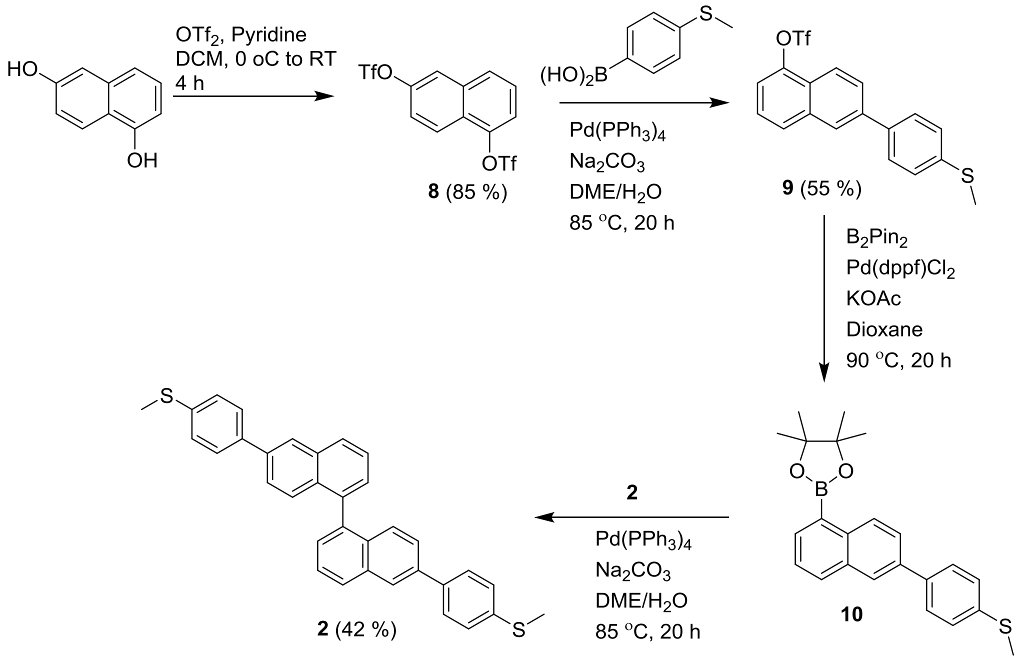

Synthesis of naphthalene-1,6-diyl bis(trifluoromethanesulfonate) (8)

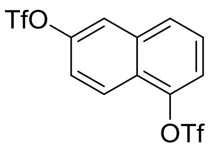

In a flame-dried 100 mL Schlenk flask, naphthalene-1,6-diol (1.5 g, 9.37 mmol) was dissolved in dry DCM (15 mL). The solution was cooled down to 0 °C and anhydrous pyridine (2.3 mL, 28.11 mmol) was added drop-wise, followed by addition of trifluoromethanesulfonic anhydride (3.46 mL, 20.81 mmol). Then, the reaction mixture was refluxed for overnight. When completed, the mixture was cooled to room temperature and diluted with DCM. 1N HCl solution was

carefully added drop-wise and the layers were separated. The organic layer was washed with saturated $NaHCO_3$, brine, and dried over anhydrous $MgSO_4$. The solvent was removed by rotary evaporation, then the title compound was purified by column chromatography on silica gel hexanes: ethyl acetate mixture (*v:v,* 30:70) giving compound **8** as a pale-yellow oil (2.48 g, 85% yield). ^{1}H NMR (500 MHz, $CDCl_3$) δ 8.19 (d, *J* = 9.3 Hz, 1H), 7.92 (d, *J* = 8.2 Hz, 1H), 7.86 (d, *J* = 2.3 Hz, 1H), 7.63 (t, *J* = 8.0 Hz, 1H), 7.59 – 7.53 (m, 2H).; ^{13}C NMR (126 MHz, $CDCl_3$) δ 148.3, 145.7, 135.2, 128.8, 127.6, 125.7, 124.3, 121.9, 120.2 (d, J_{CF} = 9.2 Hz), 119.8, 119.4, 117.6 (d, J_{CF} = 9.2 Hz).; ^{19}F NMR (471 MHz, $CDCl_3$) δ -72.70, -73.18. HRMS (CI) *m/z* = 424.9591 $[M+H]^+$; calculated for $[C_{12}H_6F_6O_6S_2]^+$ 424.9588.

Synthesis of 6-(4-(methylthio)phenyl)naphthalen-1-yl trifluoromethanesulfonate (9)

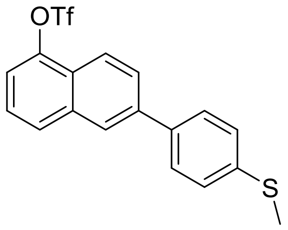

A mixture of **8** (1.0 g, 2,35 mmol), 4-(methylthio)phenylboronic acid (434 mg, 2.58 mmol), K_2CO_3 (974 mg, 7.05 mmol) were suspended in a mixture of toluene/water (11 mL/4 mL) and the mixture was degassed by three freeze-pump-thaw cycles. $Pd(PPh_3)_4$ (82 mg, 0.07 mmol) was added under a flow of nitrogen and the reaction mixture was stirred at 85°C overnight. The mixture was allowed to cool to room temperature and all the volatilities were removed. Then, the residue was extracted with dichloromethane and washed with brine and dried over anhydrous magnesium sulfate, filtered and the solvent was removed. The crude product was purified by column chromatography over silica gel with hexanes: ethyl acetate mixture (*v:v,* 80:20) as eluent to afford **9** as a white solid (517 mg, 55 % yield). ^{1}H NMR (500 MHz, $CDCl_3$) δ 8.14 (d, *J* = 8.8 Hz, 1H), 8.07 (d, *J* = 1.5 Hz, 1H), 7.94 – 7.87 (m, 2H), 7.69 – 7.62 (m, 2H), 7.51 (t, *J* = 7.9 Hz, 1H), 7.47 – 7.43 (m, 1H), 7.42 – 7.36 (m, 2H), 2.55 (s, 3H).; ^{13}C NMR (126 MHz, $CDCl_3$) δ 145.9, 139.8, 138.9, 136.9, 135.5, 128.9, 127.9, 127.6, 127.1, 125.9, 125.6, 125.6, 121.7, 117.9, 15.94.; ^{19}F NMR (471 MHz, $CDCl_3$) δ -73.27.

Synthesis of 4,4,5,5-tetramethyl-2-(6-(4-(methylthio)phenyl)naphthalen-1-yl)-1,3,2-dioxaborolane (10)

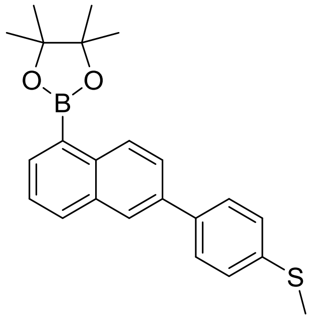

A mixture of **9** (257 mg, 0.65 mmol), bis(pinacolato)diboron (360 mg, 1.419 mmol), KOAc (380 mg, 3.87 mmol) and anhydrous 1,4-dioxane (10 mL) were degassed by three freeze-pump-thaw cycles. [1,1′-Bis(diphenylphosphino)ferrocene]dichloropalladium(II) (48 mg, 0.06 mmol) was added under a flow of nitrogen and the

reaction mixture was heated to 80 °C overnight under an atmosphere of nitrogen. The mixture was allowed to cool to room temperature and the solvent was removed to isolate the crude intermediate which was used without further purification.

Synthesis of 6,6'-bis(4-(methylthio)phenyl)-1,1'-binaphthalene (2)

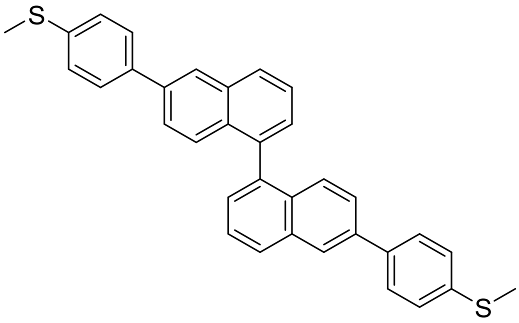

A mixture of the crude intermediate **10** (242 mg, 0.65 mmol), the naphthalene triflate **9** (257 mg, 0.65 mmol), Na_2CO_3 (545 mg, 5.16 mmol), dimethoxyethane (6 mL) and water (6 mL) was degassed by three freeze-pump-thaw cycles. $Pd(PPh_3)_4$ (37 mg, 0.03 mmol) was added under a flow of nitrogen and the reaction mixture was stirred at 85 °C overnight. The mixture was allowed to cool to room temperature and the organic solvents removed. The residue was extracted with dichloromethane. The organic extract was washed with brine, dried over anhydrous magnesium sulfate, filtered and the solvent was removed. The crude material was purified by column chromatography over silica gel with *n*-hexane: ethyl acetate mixture (*v:v,* 85:15) as eluent, followed by recrystallisation from ethyl acetate to afford the title compound **4** as white solid (135 mg, 42 %). ^{1}H NMR (500 MHz, $CDCl_3$) δ 8.13 (d, *J* = 1.3 Hz, 2H), 8.01 (d, *J* = 8.2 Hz, 2H), 7.63 (t, *J* = 8.4 Hz, 6H), 7.57 – 7.45 (m, 6H), 7.36 (d, *J* = 8.4 Hz, 4H), 2.54 (s, 6H).^{13}C NMR (126 MHz, $CDCl_3$) δ 138.5, 138.1, 138.0, 137.9, 134.1, 132.2, 128.4, 128.1, 127.9, 127.4, 127.2, 126.2, 125.8, 125.6, 16.1. HRMS (CI) *m/z* = 499.1512 $[M+H]^+$; calculated for $[C_{34}H_{27}S_2]^+$ 499.1554.

1.3. Synthesis of 3

The synthesis of 3 was performed following the route designed for **1**, but starting from 6-methoxy-3,4-dihydronaphthalen-1-one, delivering the ditriflate **14** which was coupled to 2-(methylthio)phenylboronic acid under Suzuki conditions to yield **3**. Compounds **11** to **14** were synthesised according to a previously described procedure.[1]

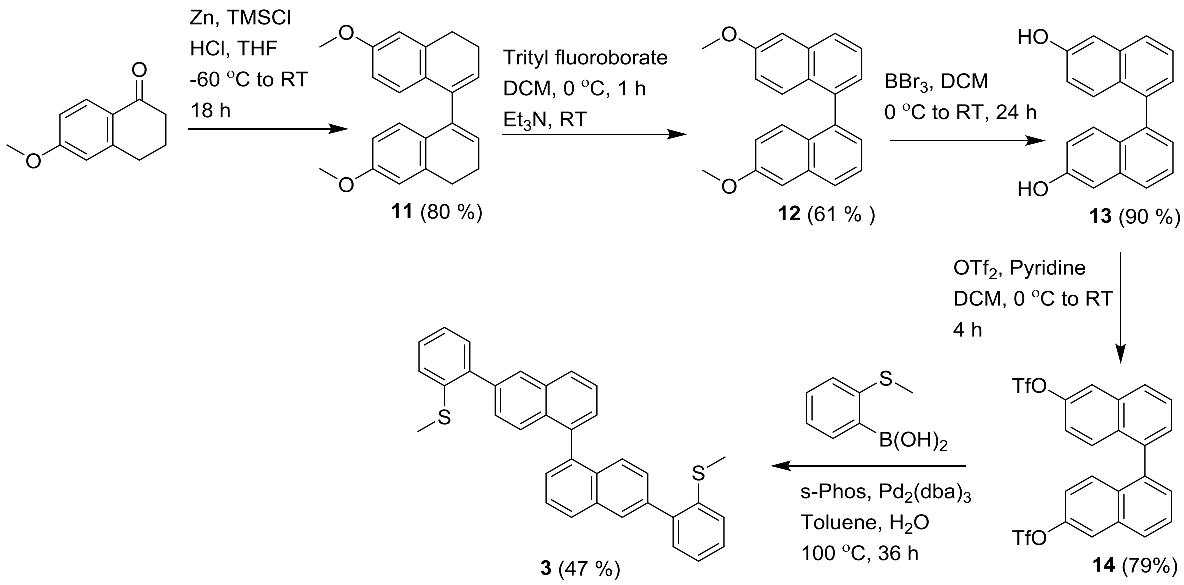

Synthesis of 6,6'-bis(2-(methylthio)phenyl)-1,1'-binaphthalene (3)

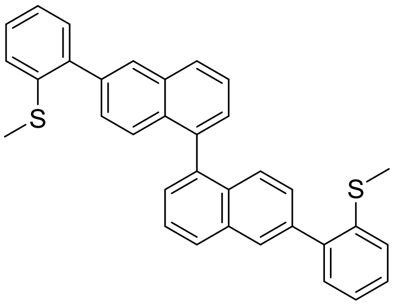

A mixture of compound **14** (417 mg, 0.76 mmol), (2-(methylthio)phenyl)boronic acid (382 mg, 2.27 mmol), K_3PO_4 (0.963 g, 4.55 mmol), and toluene/ water (10 mL/ 1 mL) was degassed by three freeze-pump-thaw cycles. $Pd_2(dba)_3$ (36 mg, 0.03 mmol) and S-Phos (31 mg, 0.08 mmol) were added under a flow of nitrogen and the resulting solution was heated to 100 °C for 36 h. The mixture was allowed to cool to room temperature and the solvents were removed under vacuum. The organic part was extracted in DCM and the organic extract was washed with brine, dried over anhydrous magnesium sulfate, filtered and the solvent was removed. The crude material was purified by flash column chromatography over silica gel with hexanes: ethyl acetate mixture (*v:v,* 95:5) as eluent, followed by recrystallisation from $CHCl_3$/pentane to give the title compound **3** as white solid (178 mg, 47 %). ^{1}H NMR (500 MHz, $CDCl_3$) δ 7.98 (dd, *J* = 4.7, 3.2 Hz, 4H), 7.65 – 7.59 (m, 2H), 7.57 – 7.52 (m, 4H), 7.42 (dd, *J* = 8.7, 1.7 Hz, 2H), 7.39 – 7.34 (m, 2H), 7.32 (dd, *J* = 7.2, 1.6 Hz, 4H), 7.25 – 7.19 (m, 2H), 2.38 (s, 6H). ^{13}C NMR (126 MHz, $CDCl_3$) δ 140.9, 138.5, 138.2, 137.5, 133.5, 132.3, 130.5, 128.6, 128.4, 128.3 (br), 127.9, 126.5, 125.9, 125.5, 125.0, 16.3. One resonance for a sp^2 carbon is unobserved, possibly convoluted with another resonance in the broad signal at 128.3 ppm. HRMS (CI) *m/z* = 499.1527 $[M+H]^+$; calculated for $[C_{34}H_{27}S_2]^+$ 499.1554.

2. NMR Spectra

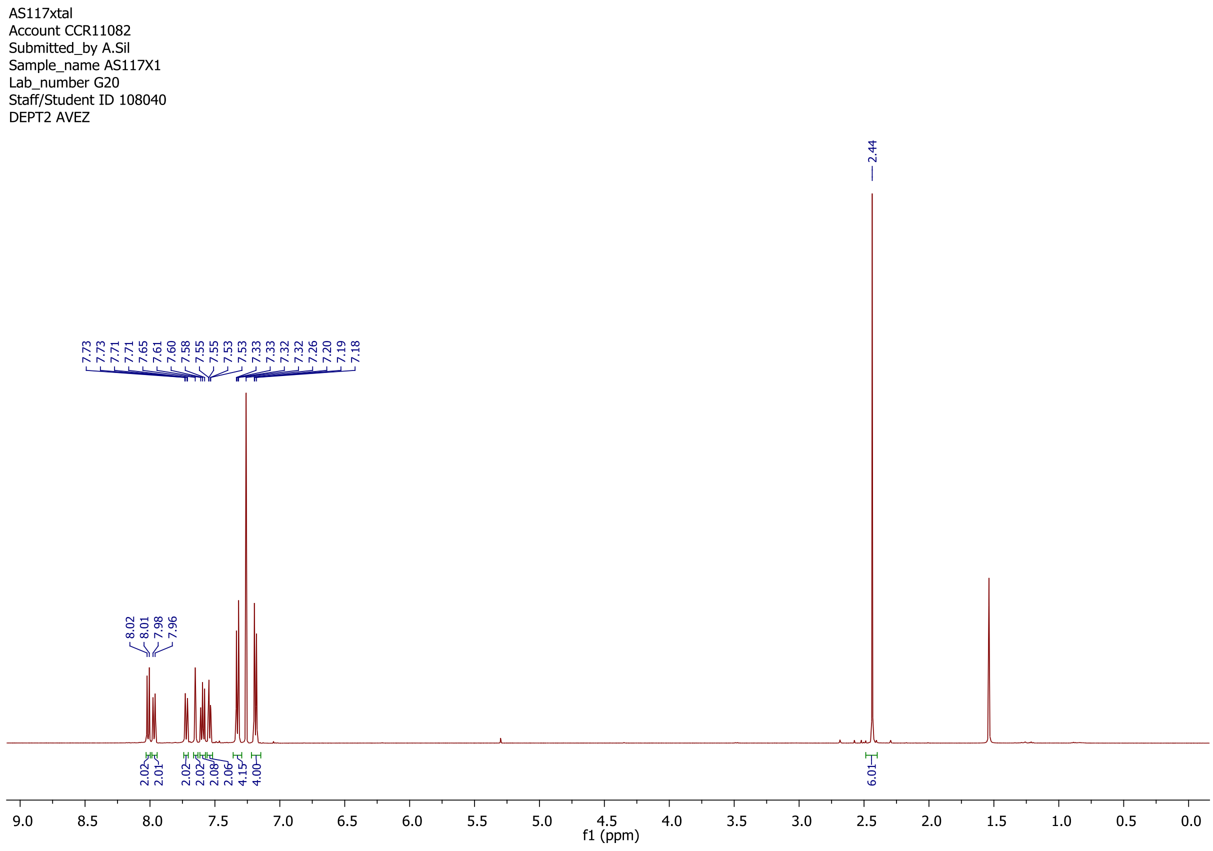

Figure S1: ^{1}H NMR (500 MHz, $CDCl_3$) of **1**

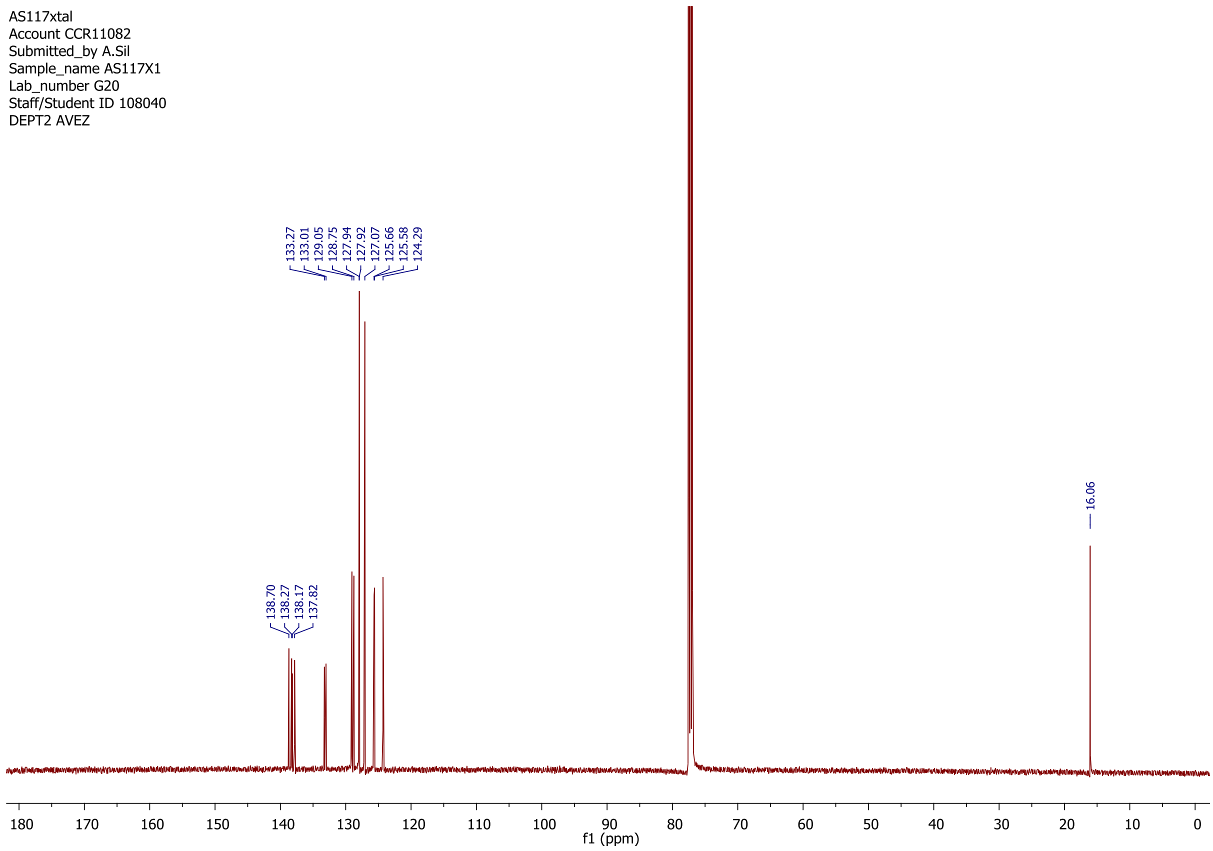

Figure S2: ^{13}C NMR (126 MHz, $CDCl_3$) of **1**

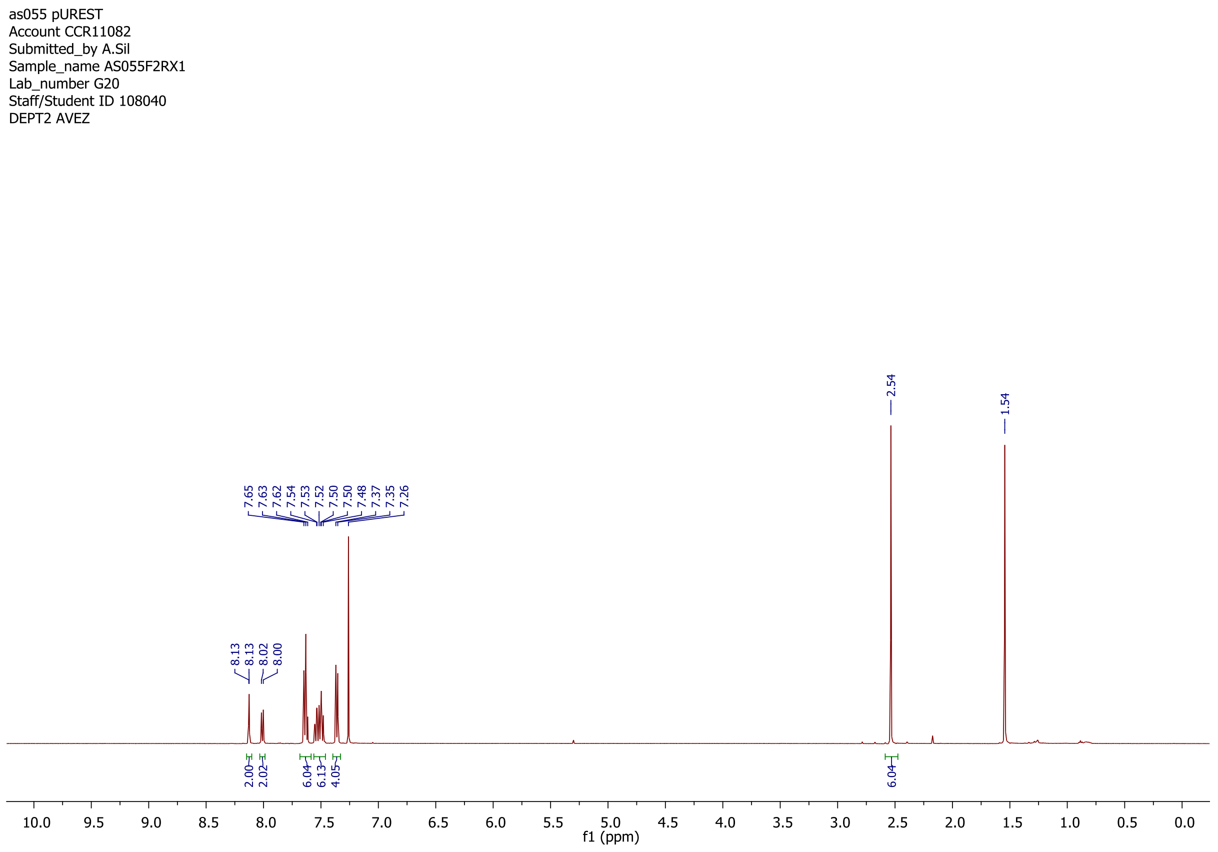

Figure S3: ^{1}H NMR (500 MHz, $CDCl_3$) of **2**

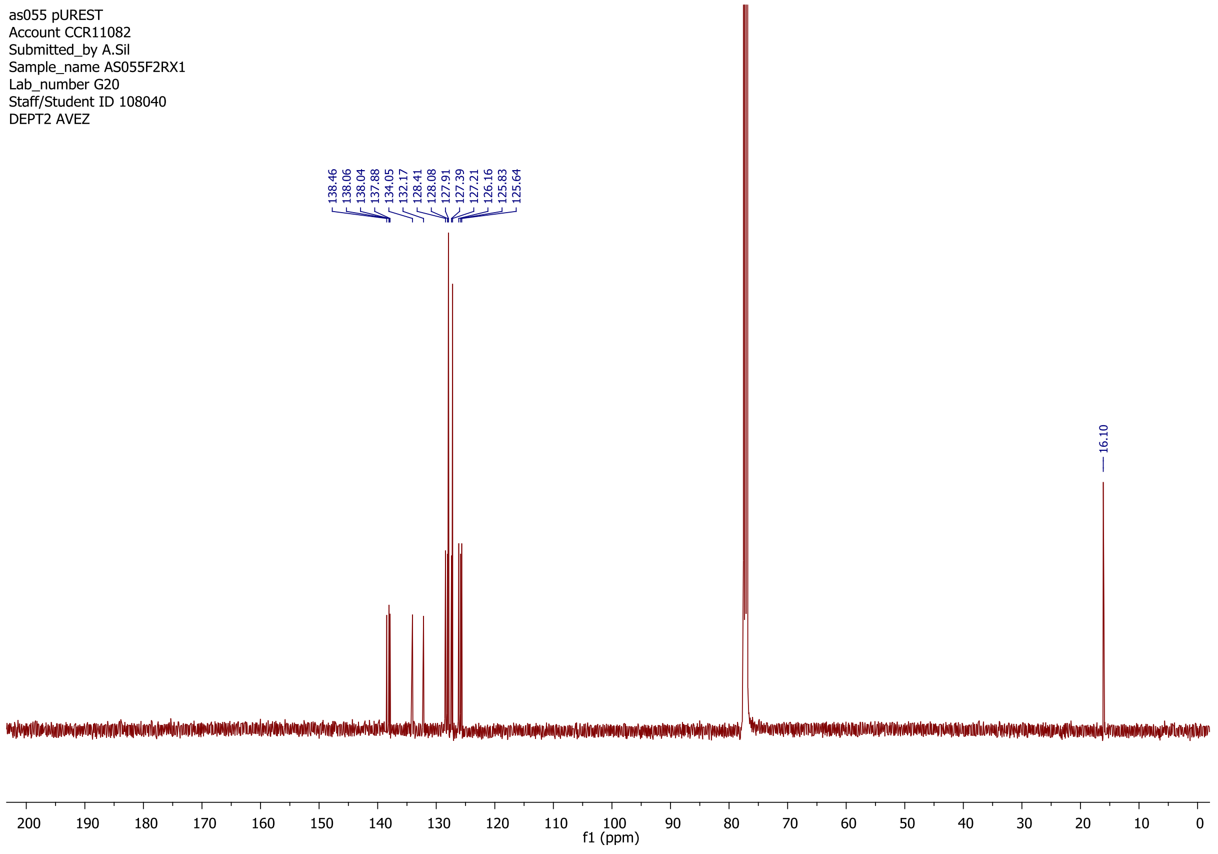

Figure S4: ^{13}C NMR (126 MHz, $CDCl_3$) of **2**

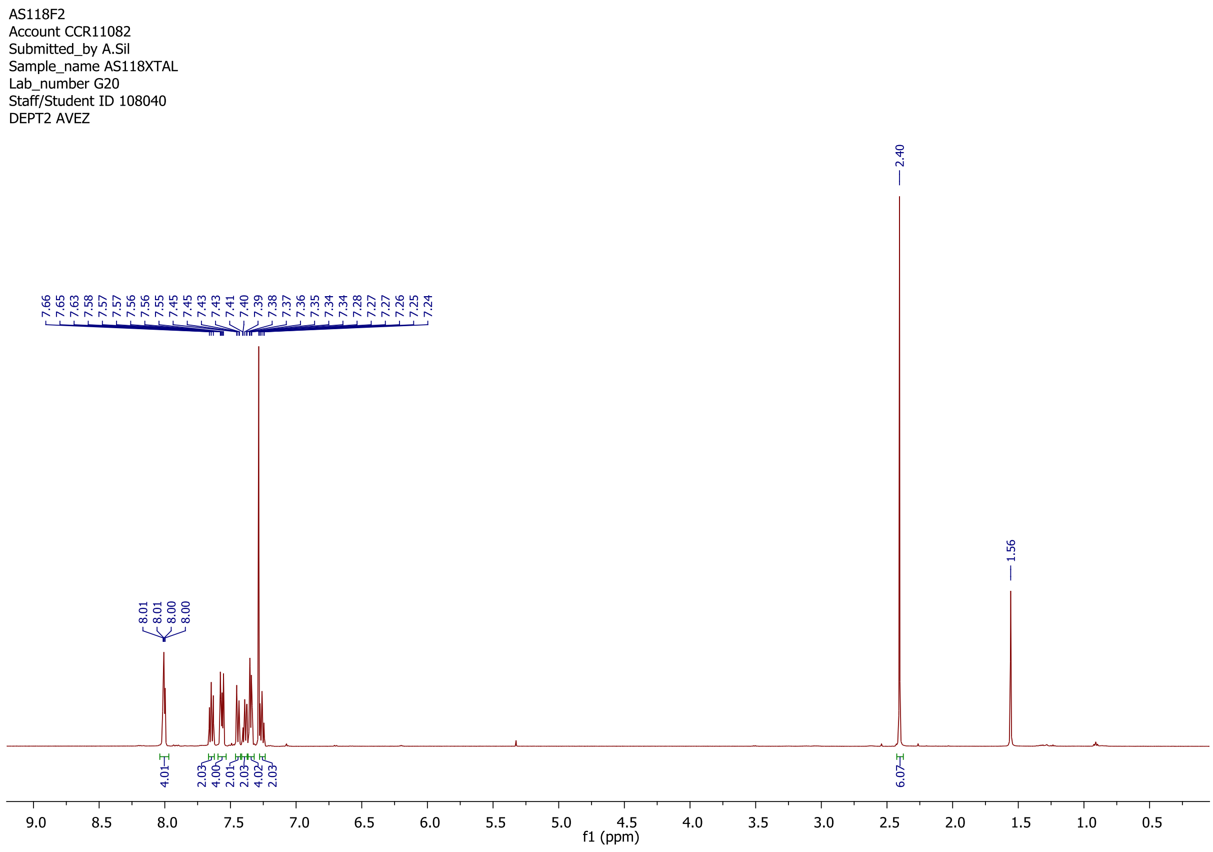

Figure S5: ^{1}H NMR (500 MHz, $CDCl_3$) of **3**

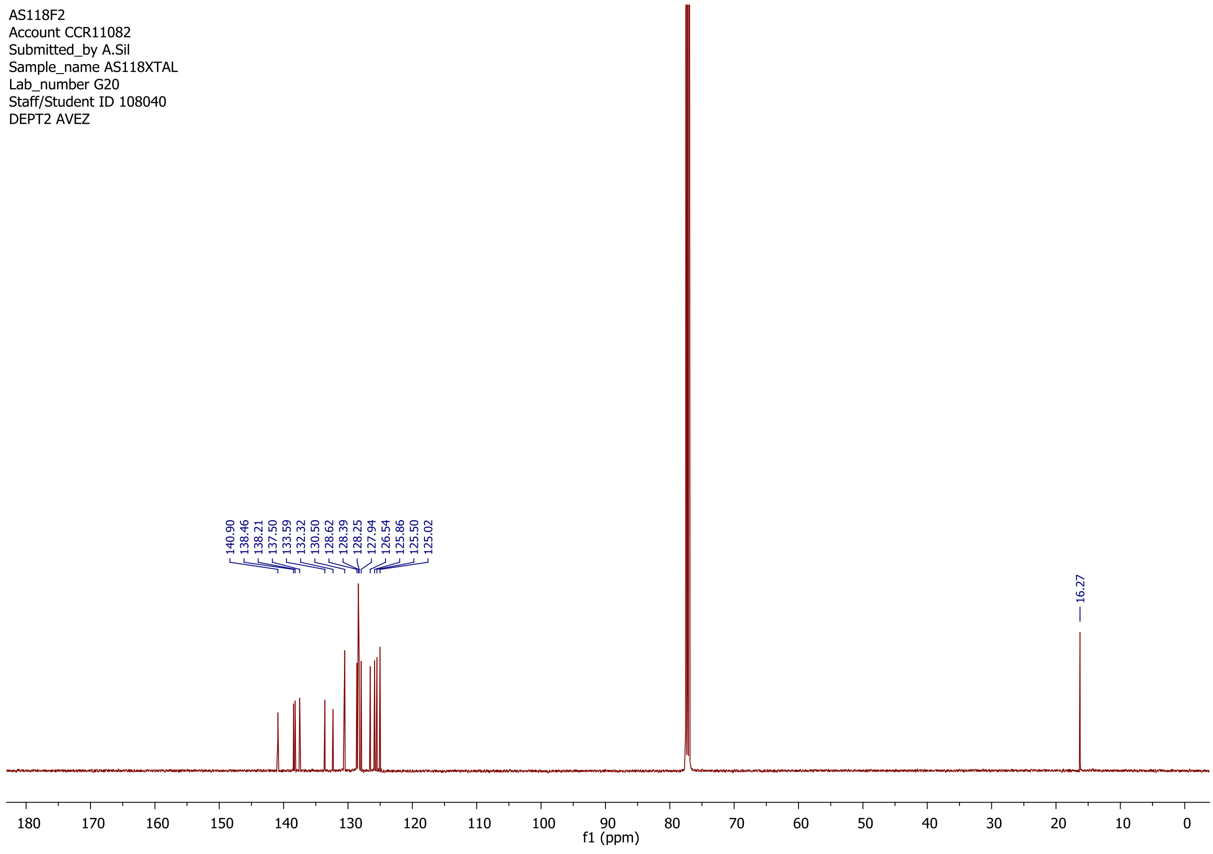

Figure S6: ^{13}C NMR (126 MHz, $CDCl_3$) of **3**

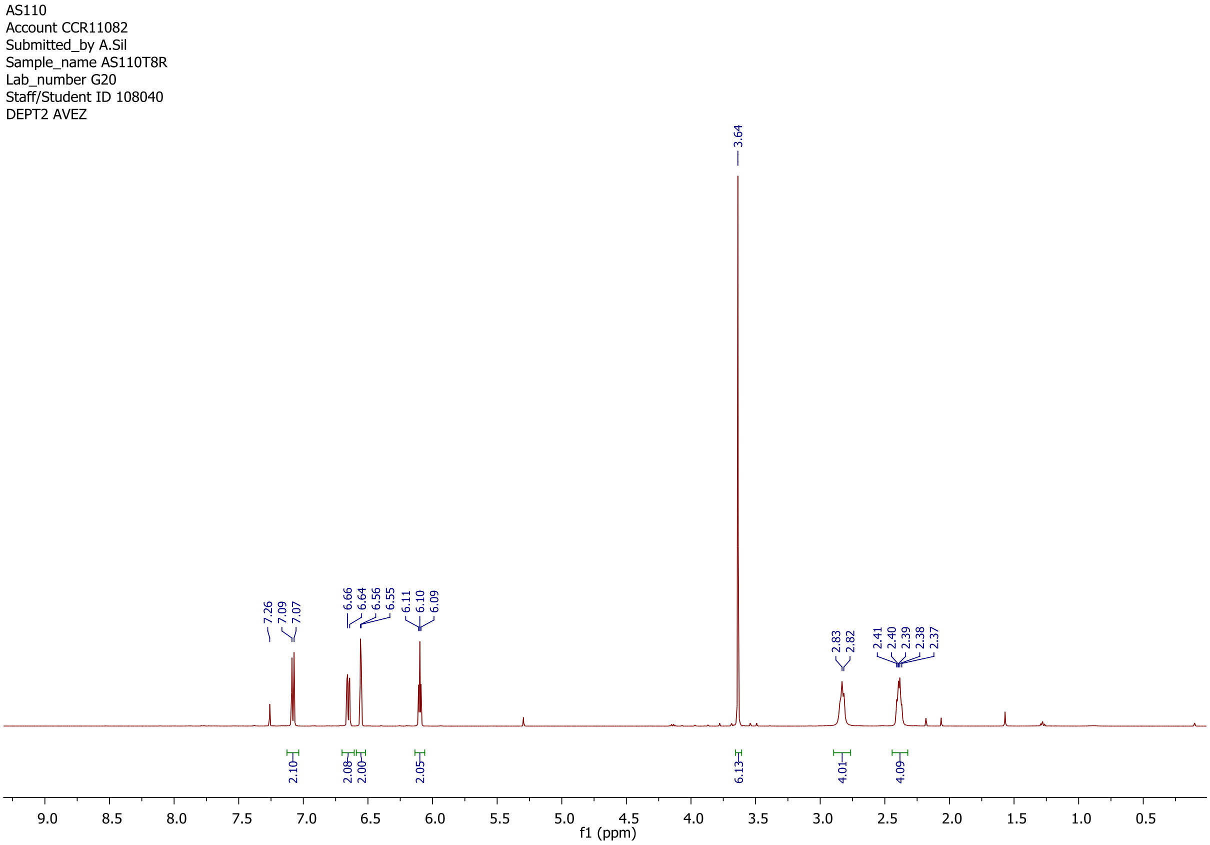

Figure S7: ^{1}H NMR (500 MHz, $CDCl_3$) of **4**

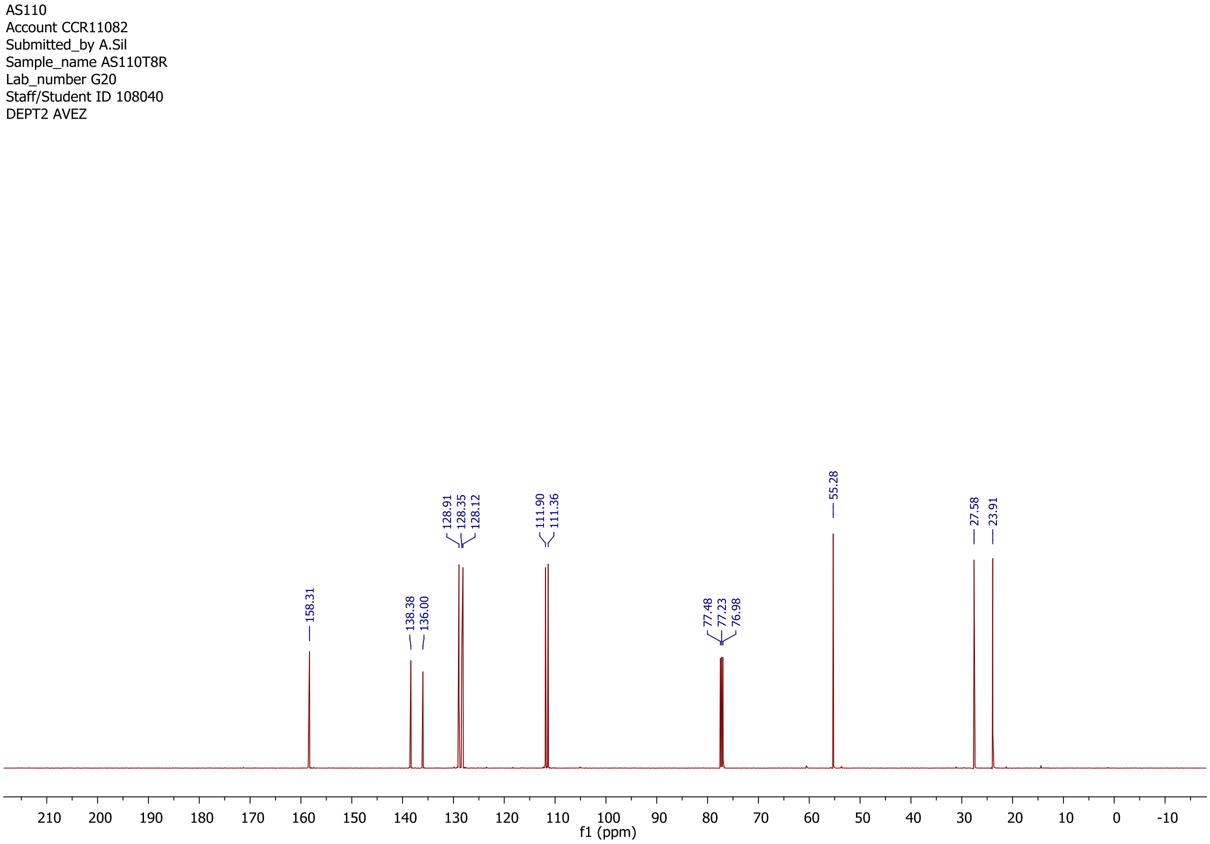

Figure S8: ^{13}C NMR (126 MHz, $CDCl_3$) of **4**

AS112
Account CCR11082
Submitted_by A.Sil
Sample_name as112_1
Lab_number G20
Staff/Student ID 108040
DEPT2 AVEZ

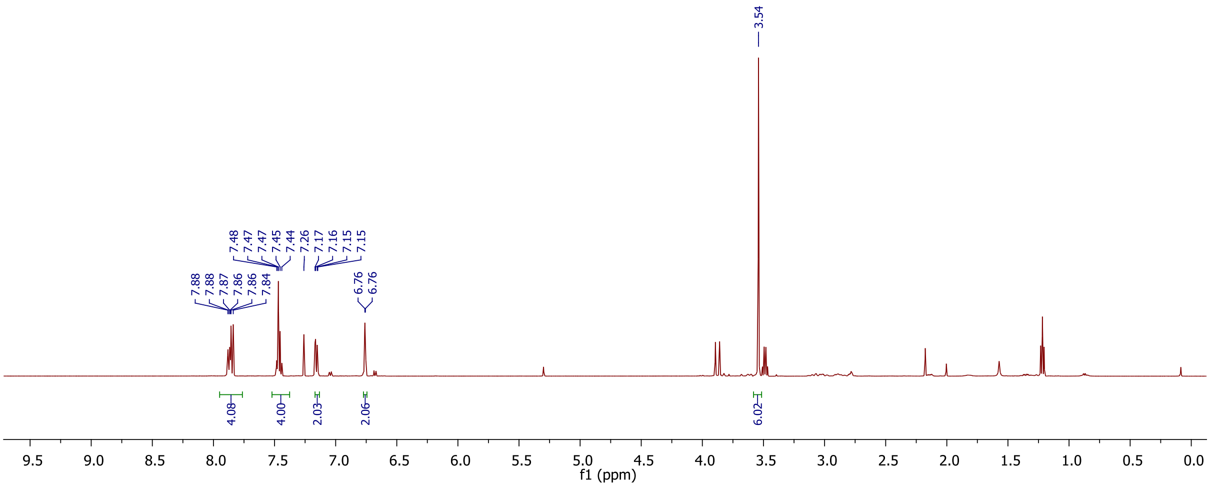

Figure S 9: ^{1}H NMR (500 MHz, $CDCl_3$) of **5**

AS112
Account CCR11082
Submitted_by A.Sil
Sample_name AS112_2
Lab_number G20
Staff/Student ID 108040
DEPT2 AVEZ

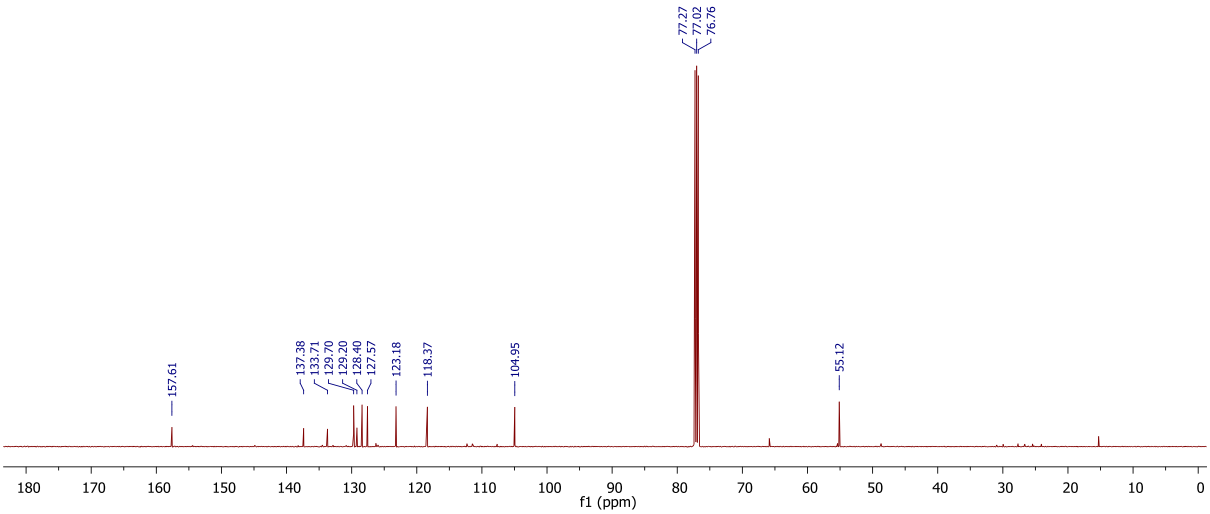

Figure S10: ^{13}C NMR (126 MHz, $CDCl_3$) of **5**

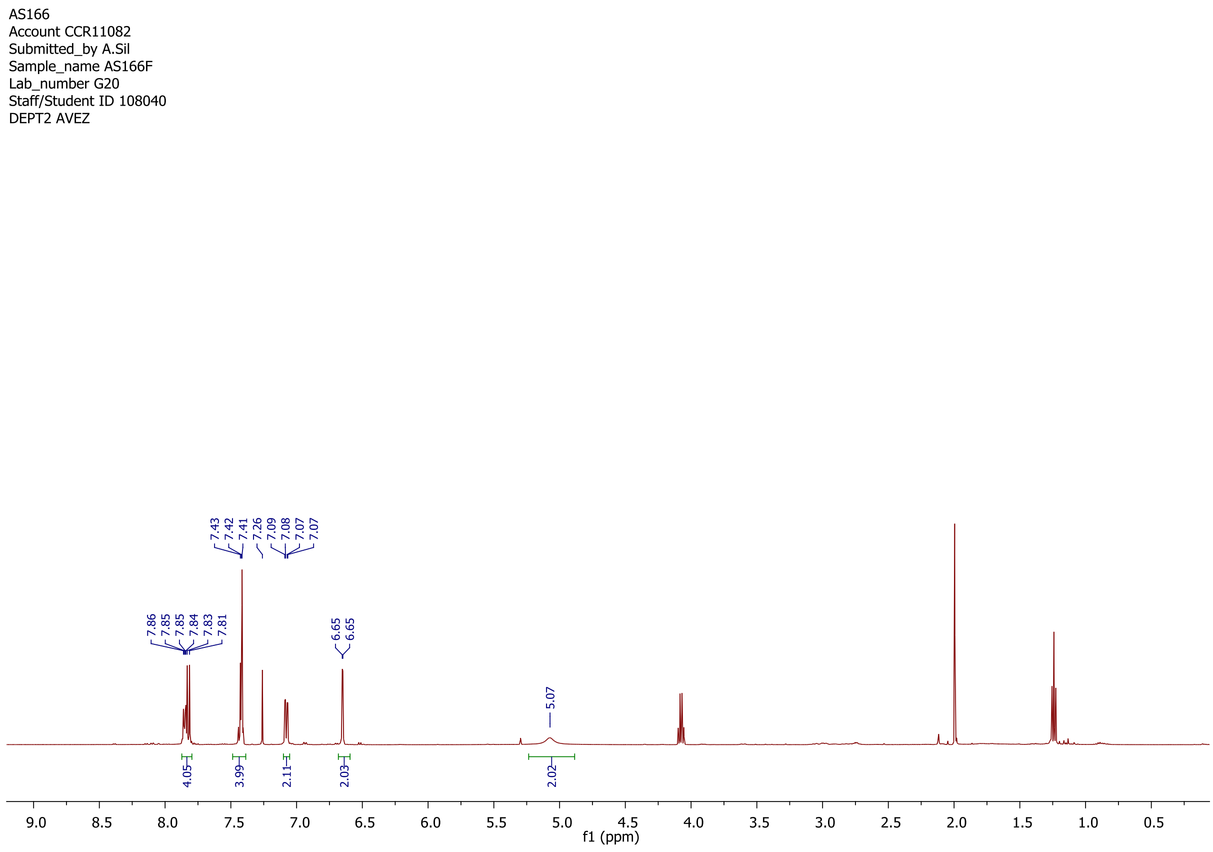

Figure S11: ^{1}H NMR (500 MHz, $CDCl_3$) of **6**

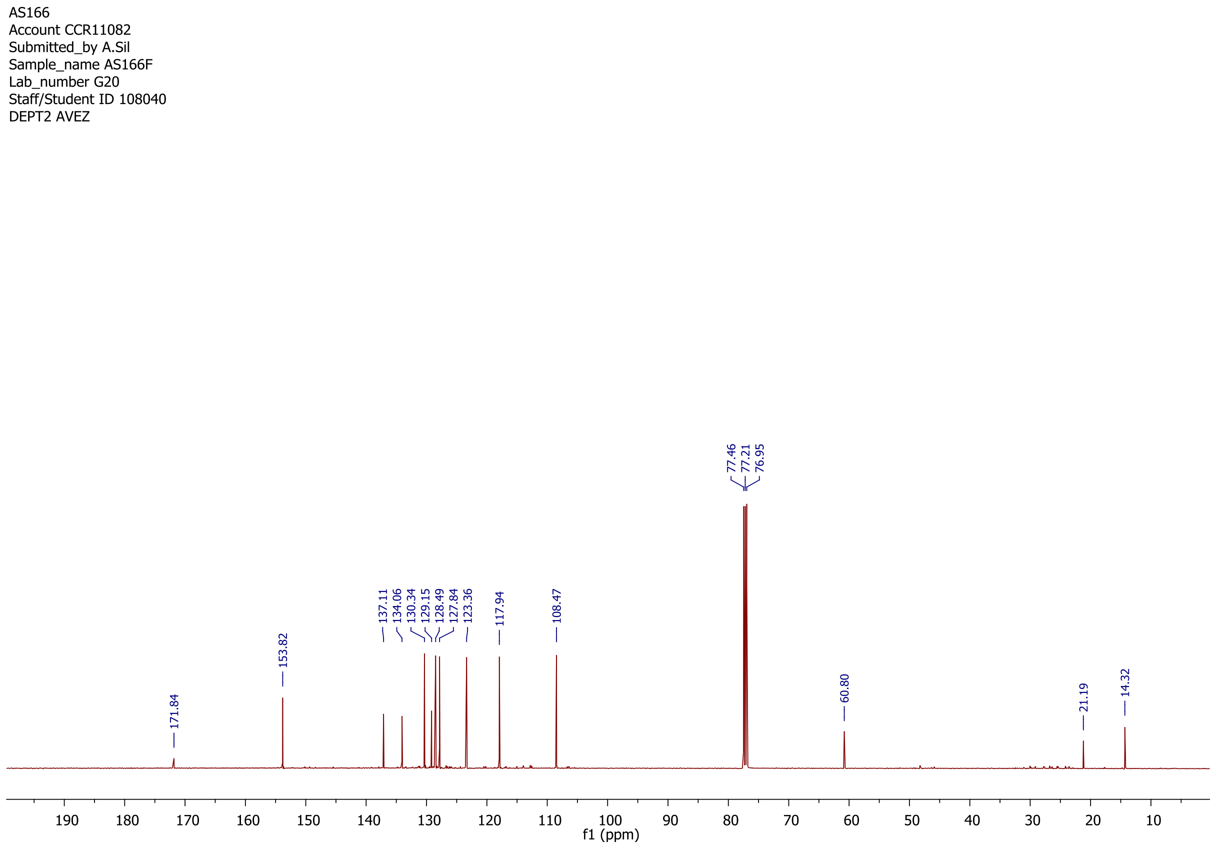

Figure S12: ^{13}C NMR (126 MHz, $CDCl_3$) of **6**

AS116F
Account CCR11082
Submitted_by A.Sil
Sample_name AS116F
Lab_number G20
Staff/Student ID 108040
DEPT2 AVEZ

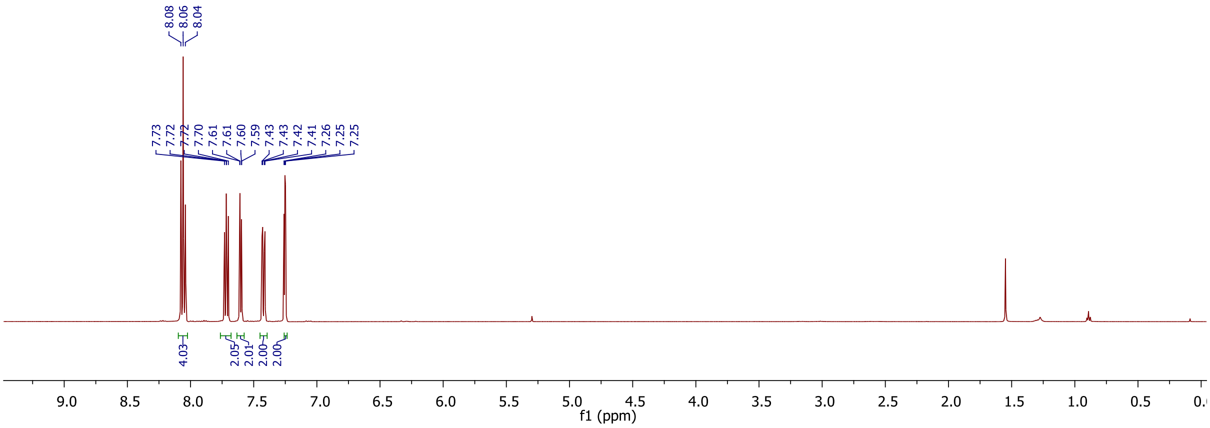

Figure S13: ^{1}H NMR (500 MHz, $CDCl_3$) of **7**

AS116F
Account CCR11082
Submitted_by A.Sil
Sample_name AS116F
Lab_number G20
Staff/Student ID 108040
DEPT2 AVEZ

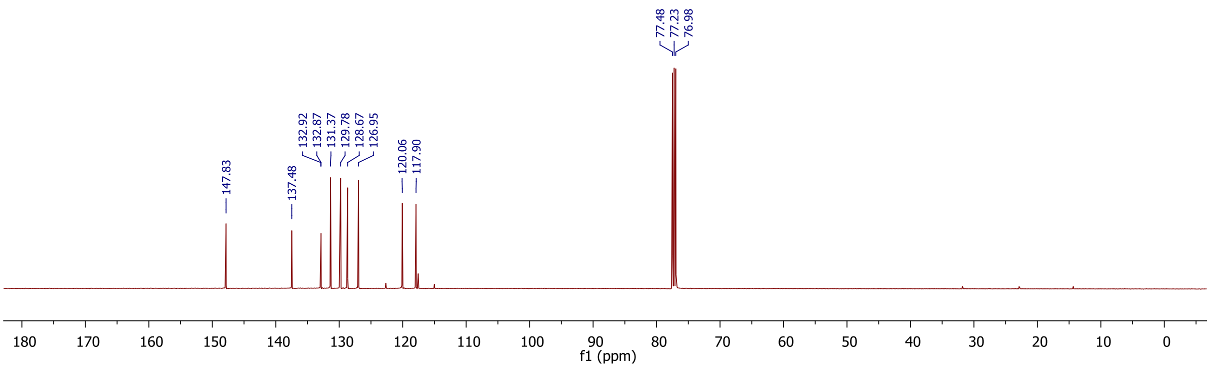

Figure S14: ^{13}C NMR (126 MHz, $CDCl_3$) of **7**

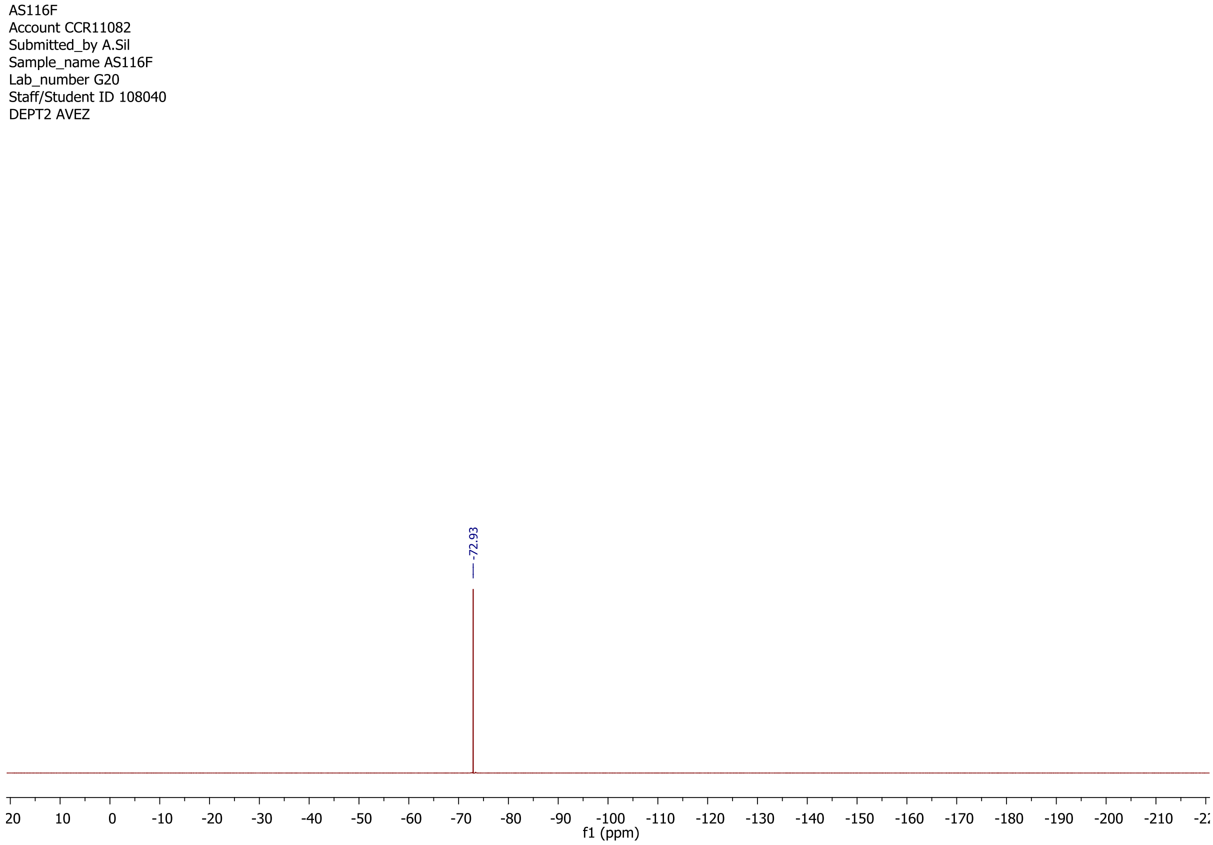

Figure S15: ^{19}F NMR (471 MHz, $CDCl_3$) of **7**

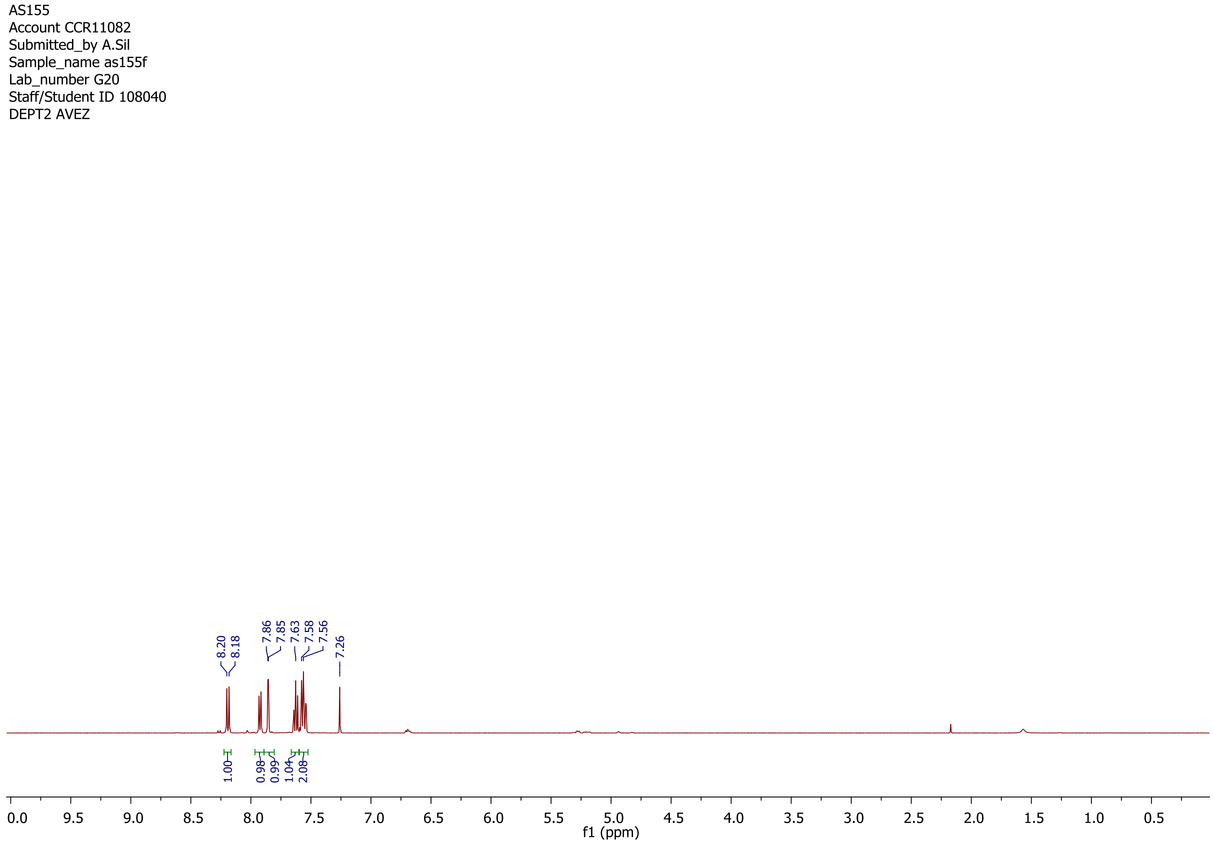

Figure S16: ^{1}H NMR (500 MHz, $CDCl_3$) of **8**

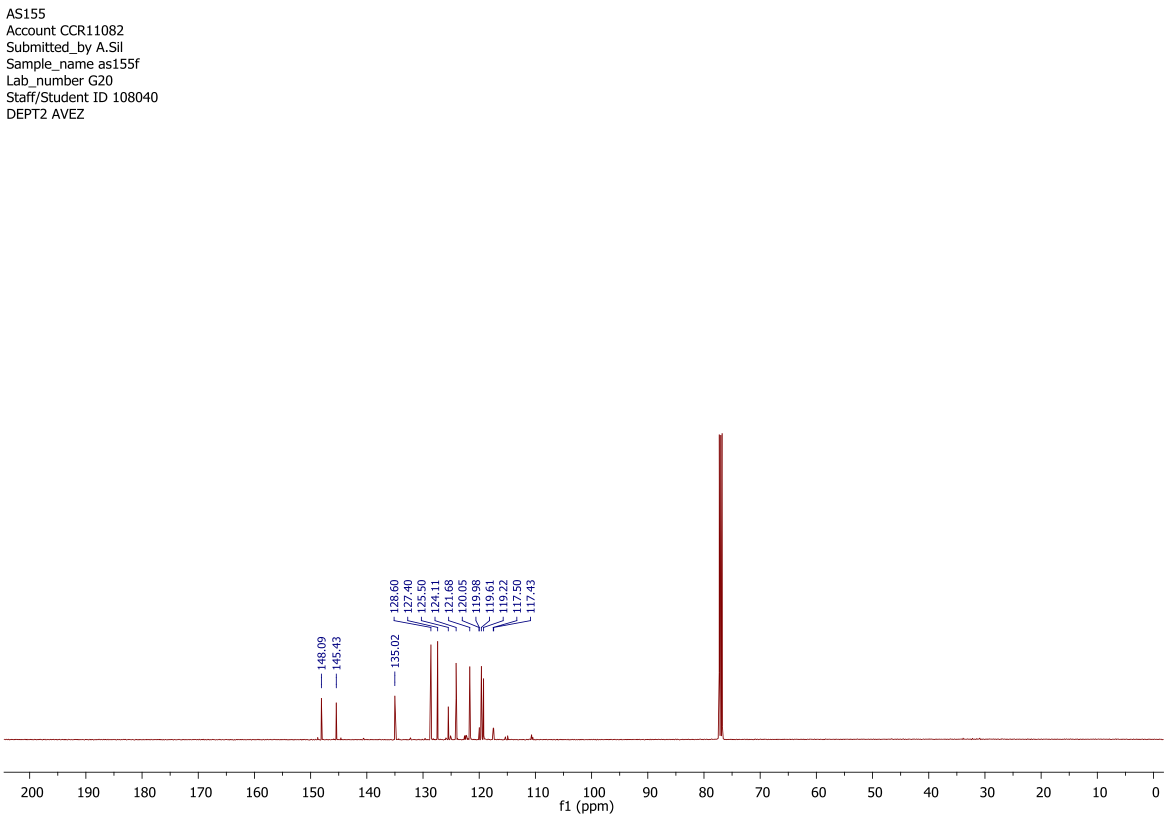

Figure S17: ^{13}C NMR (126 MHz, $CDCl_3$) of **8**

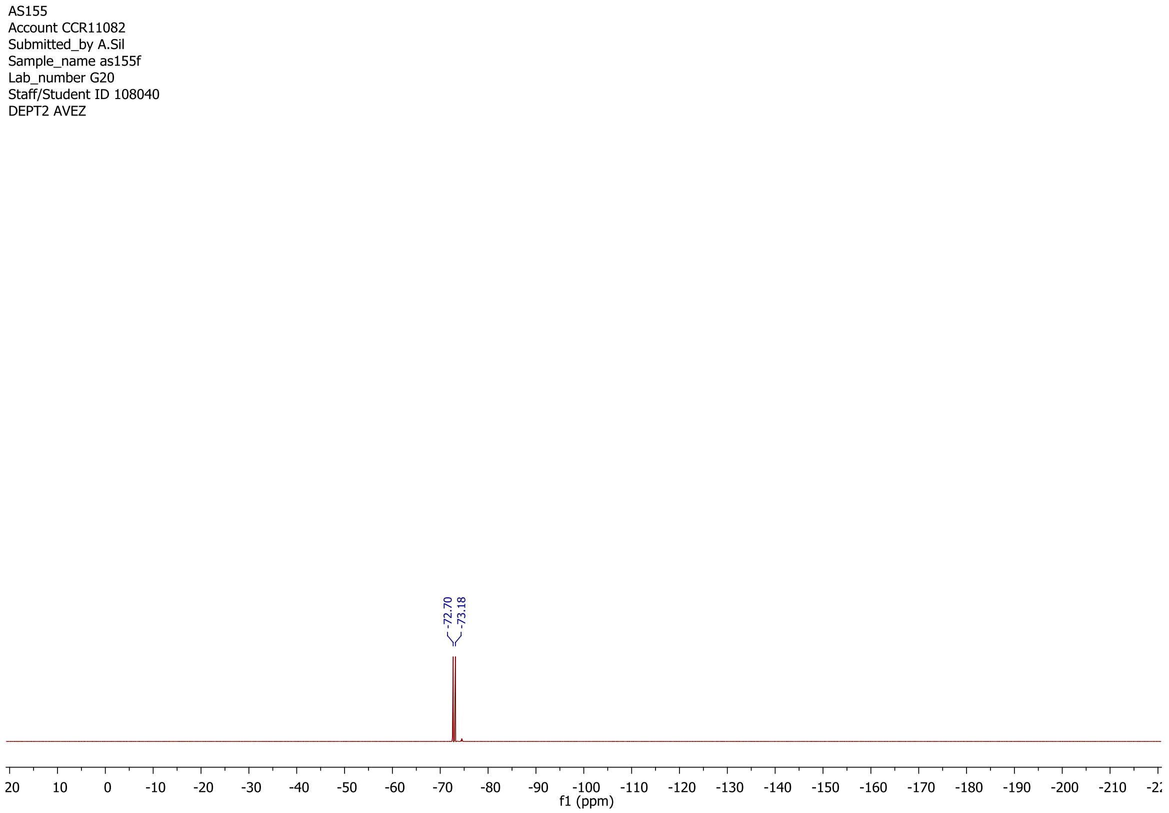

Figure S18: ^{19}F NMR (471 MHz, $CDCl_3$) of **8**

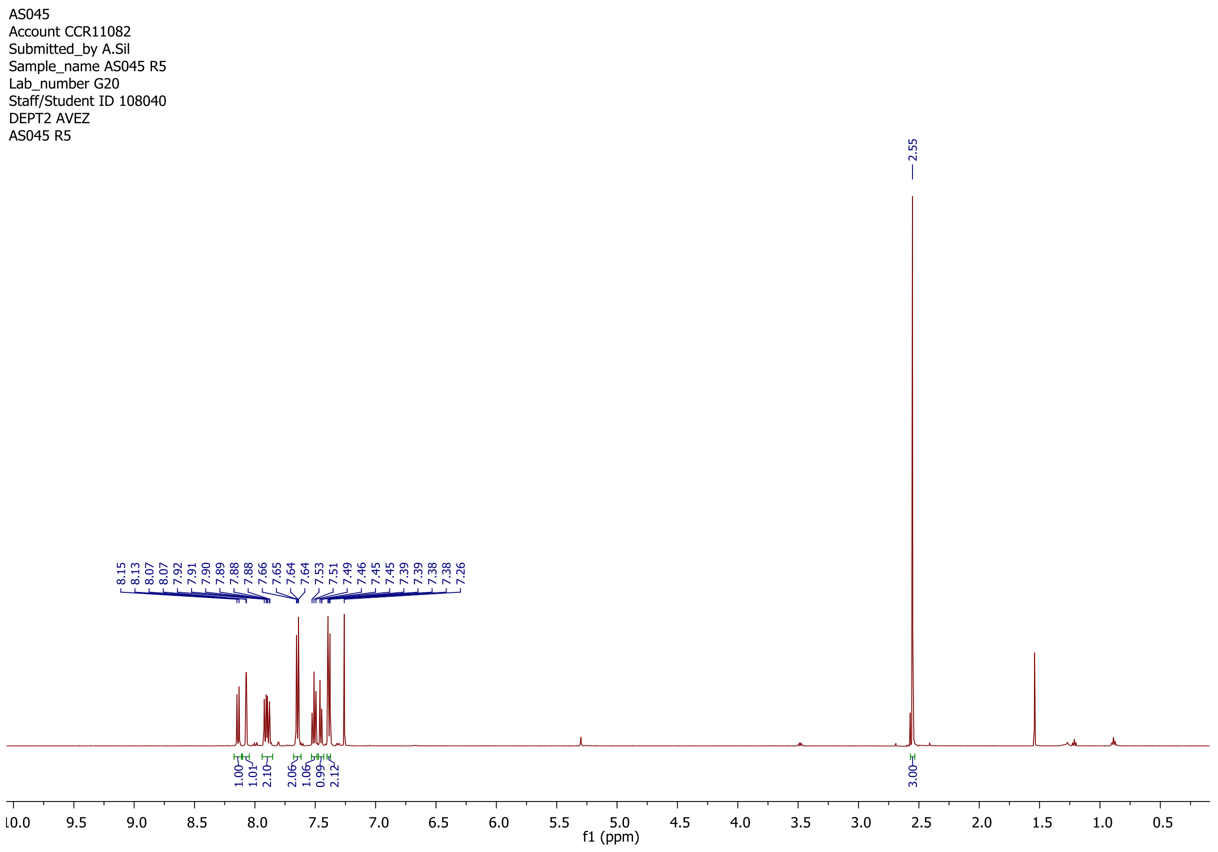

Figure S19: ^{1}H NMR (500 MHz, $CDCl_3$) of **9**

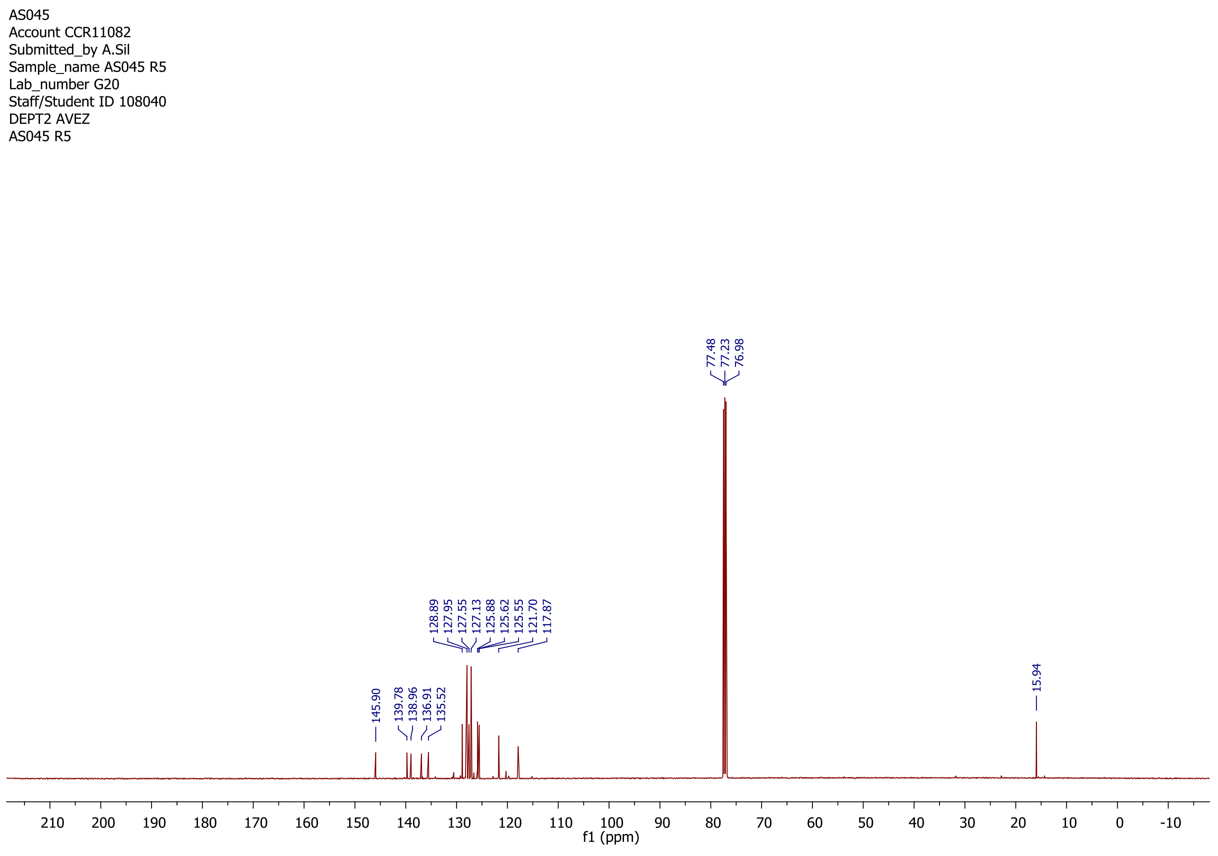

Figure S20: ^{13}C NMR (126 MHz, $CDCl_3$) of **9**

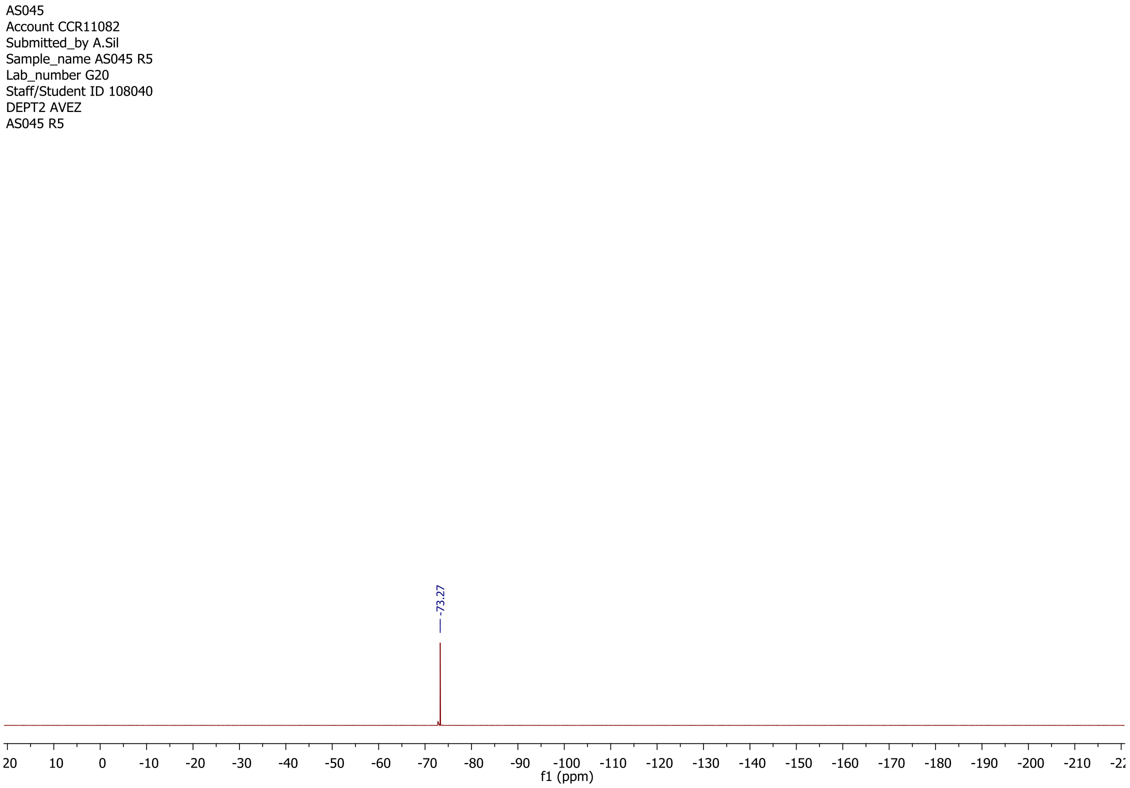

Figure S21: ^{19}F NMR (471 MHz, $CDCl_3$) of **9**

3. Details on STMBJ measurements

We used a modified Keysight 5500 scanning tunnelling microscope (Figure S22), equipped with a Femto DLPCA-200 current amplifier. Piezo voltage and tip bias are imposed by an Arbitrary Wavefunction Generator (AWG, Keysight 33522B), and signals are recorded with a National Instrument PXI system (24-bit PXI-4464 DAQ, PXIe-1062Q chassis, PXIe-PCIe8381 interface). The Z piezo voltage signal is fed directly into the STM controller, that has an in-built voltage amplifier to convert the $\pm 10\ V$ signal of the AWG to the $\pm 215\ V$ that drive the Z piezoelectric transducer. In this study, we employed a $100\ k\Omega$ resistor after the function generator, to reduce the bias applied to junctions of conductance $> 0.1\ G_0$. As discussed in our recent publication,[2] the resistor prevents overload of the amplifier and causes an almost complete junction bias drop at values of conductance $>\ G_0$, extending the measurement window to ~8 orders of magnitude (from $\cong 10^2 G_0$ to $\cong 10^{-6} G_0$) and protecting the fragile Au atomic contact from electromigration.

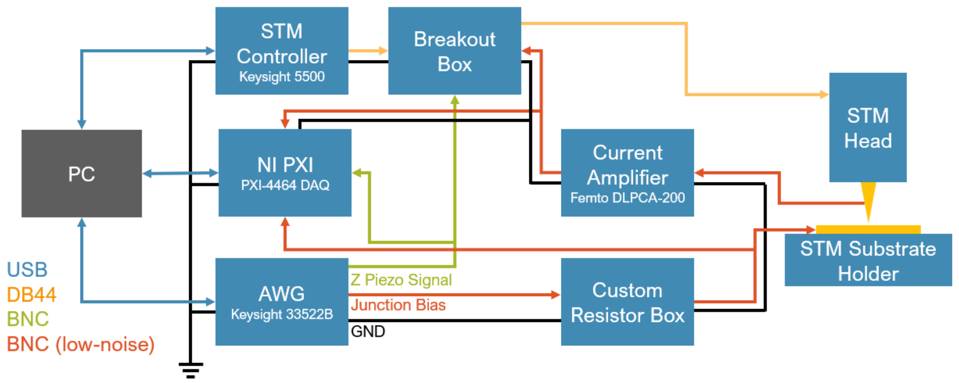

Figure S22: Schematics of the modified Keysight 5500 STM used in this study

The STMBJ experiments in this study have been performed using an Au tip cut from a spool of Au wire (99.998%, ThermoFisher Scientific PREMION), and substrates are prepared by e-beam evaporation (Korvustech HEX Tau4) of ~100 nm of Au on freshly cleaved mica (muscovite, Agar Scientific). Substrates were briefly flame-annealed with a butane torch before use. Experiments were all performed in mesitylene (1,3,5-trimethylbenzene 98+%, TCI UK) with the target molecular wire in 1 mM concentration. Further information is available in our previous publications.[3,4]

3.1. STMBJ Measurements

Bespoke software written in LabVIEW controls the instrument and acquires data. We continuously record the signal applied to the piezoelectric transducer V_Z, the junction bias V_{BIAS} and the output of the Femto amplifier V_{OUT}, at a rate of $20\ kSa/s$ and a resolution of 24 bits. The current I is calculated from V_{OUT} using the amplification factor ($10^6\ V/A$ in this study) and the junction bias is then calculated using Ohm's law $G = I/V_{BIAS}$ in units of G_0. The tip displacement is calculated from the value of V_Z using the amplification factor of the STM controller electronics (10:215 ratio) and the calibration against Au(111) atomic step height (currently $19.1\ Å/V$). A trigger/gate function cuts the continuous stream of data into single traces representative of a single cycle of tip approach and withdrawal. Each trace is then checked

by an automated algorithm, that ensures that a clean metallic junction of $G > 5G_0$ is fabricated as the tip is crashed into the substrate, and that the current decays below the noise level of the instrument (~ 50 pA) at the end of the Z ramp. The piezo is moved at a constant speed of $10\ nm/s$ in this study. All data that satisfies the conditions described earlier are then used without further selection, and compiled into histograms, density plots and correlogram as presented in the manuscript and here in the SI.

3.2. Piezo-Modulation STMBJ Measurements

In the piezo-modulation experiments, a custom ramp is applied to the piezoelectric transducer in which the tip is withdrawn in abrupt steps, followed by the modulation cycles. Data is acquired without any selection and fed into an automated algorithm for sorting. An example of a raw piezo-modulation trace is shown in Figure S23.

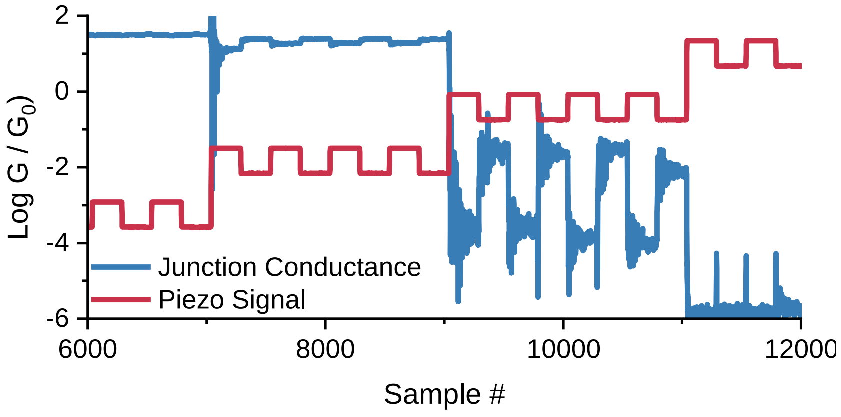

Figure S23: Example of raw piezo-modulation trace.

The sorting algorithm calculates the second derivative of the piezo signal and cuts when it goes above a threshold value (0.2 in this study, see Figure S24)Figure S24: Second derivative of the piezo signal, with threshold value in dashed line. to deliver individual slices of the modulation – each corresponding to a single set of modulation performed at a specific distance from the substrate.

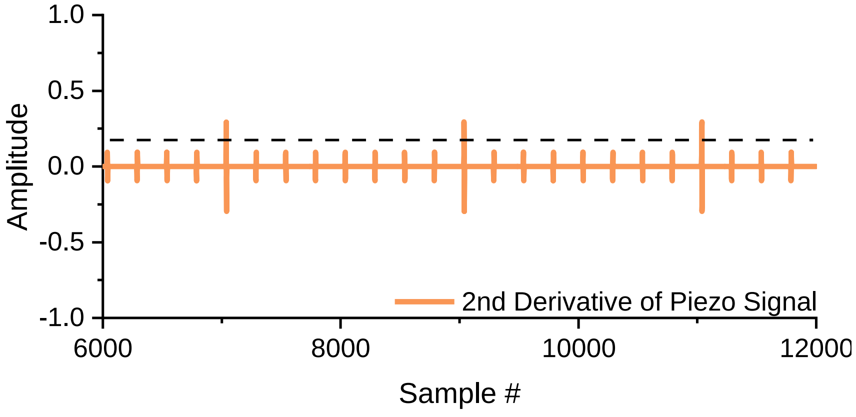

Figure S24: Second derivative of the piezo signal, with threshold value in dashed line.

The slices obtained this way are then selected by taking the average of the first and last modulation corresponding to the junction fabricated in its most extended configuration, and checks that their value falls within the range $G \pm 2\sigma$ determined by regular STMBJ experiments. This process filters out slices where the tip is in contact with the substrate, those where there is no molecule bridging the nanogap, and those where the junction did not survive the whole modulation process, leaving only the slices representative of a stable and robust molecular junction. All the slices selected by the sorting algorithm are then compiled into the 2D density maps shown in the main paper.

In this study, the sorting algorithm selected traces with the following rate:

Compound	Modulation	Amplitude	Raw Dataset #	Selected Traces #	Hitrate %
**1**	Square	7 Å	11066	2375	21.4 %
**1**	Triangular	7 Å	10521	3674	34.9 %
**1**	Triangular	9 Å	9979	2019	20.3 %

In the absence of molecules bridging the gap, we have shown previously that the modulation process results only in small changes in the noise level.[5]

3.3. Correlation Plots

The correlation plot shown in the main paper was obtained using the procedure developed by Makk *et al*.[6] In brief a histogram with 50 bins per conductance decade is calculated for each STMBJ trace. The covariance of each bin of the histogram with each of the other bins is calculated, thereby yielding the autocorrelation (autocovariance) matrix $[\Gamma_n]_{\mathrm{ij}}$

Since covariance can take any value, we normalise the matrix by dividing it by the value of the diagonal element (representing the correlation of each bin with itself) thus limiting the correlation values to $-1 \leq C_{i,j} \leq 1$. The process is repeated for all traces in the dataset, all matrices are added together, and values divided by the number of traces used. The resulting, final composite matrix is plotted as a heatmap, thereby showing the 2D distribution of covariance, with values ranging from strong correlation ($C = 1$) to strong anti-correlation ($C = -1$).

3.4. Data and Software availability

Raw data and LabVIEW Vis used for its processing can be accessed free of charge on the University of Liverpool Data Catalogue, at the DOI specified in the Data Availability section of the manuscript. Executable versions of the VIs (*e.g.* not requiring a LabVIEW license to run) are available from the authors upon request. The runtime required to run the executables can be downloaded freely from the National Instruments website.[†]

[†] https://www.ni.com [retrieved 17/07/2023]

4. Additional STMBJ Data

In addition to the plots presented in the manuscript, we show here histograms and 2D density maps (conductance *vs* electrode separation *vs* counts) for **1-3**, along with relevant experimental details in each figure caption.

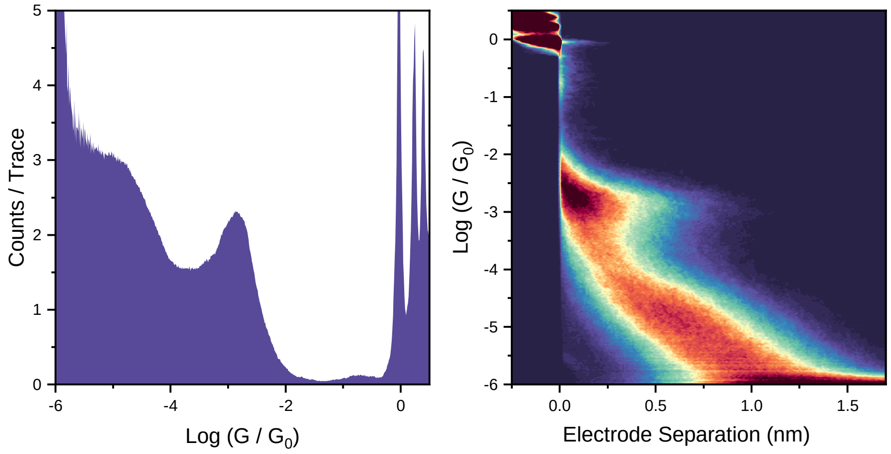

Figure S25: Conductance histogram and 2D map for **1**. Data acquired at $750\ mV$, $10\ nm\ s^{-1}$ from a 1 mM solution in mesitylene. Plots compiled from 6025 individual STMBJ traces. 100 bins per conductance decade, 100 bins per nm.

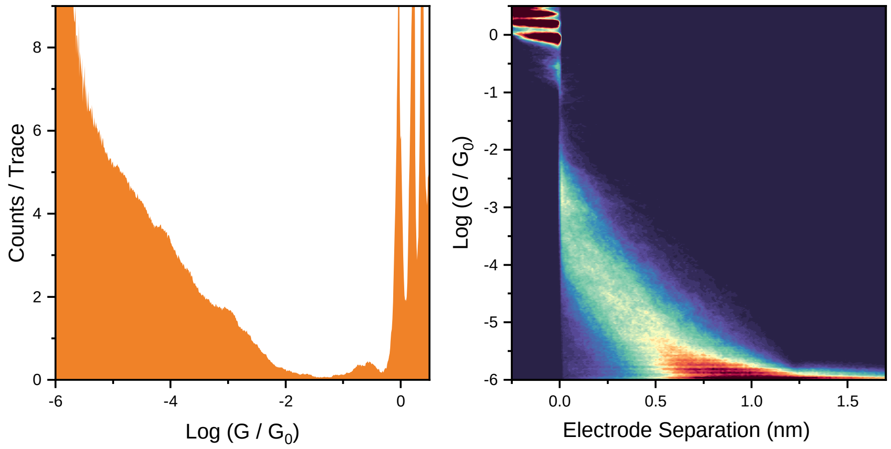

Figure S26: Conductance histogram and 2D map for **2**. Data acquired at $1\ V$, $10\ nm\ s^{-1}$ from a 1 mM solution in mesitylene. Plots compiled from 3581 individual STMBJ traces. 100 bins per conductance decade, 100 bins per nm.

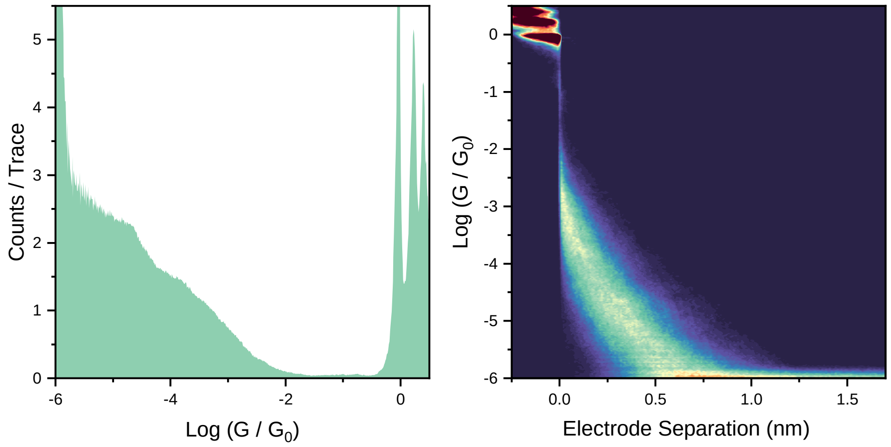

Figure S27: Conductance histogram and 2D map for **3**. Data acquired at $1\ V$, $10\ nm\ s^{-1}$ from a 1 mM solution in mesitylene. Plots compiled from 2950 individual STMBJ traces. 100 bins per conductance decade, 100 bins per nm.

5. Computational Methods

We optimized the geometries of binaphthalene using the SIESTA[7,8] implementation of density functional theory (DFT), with a double-ζ polarized basis set (DZP). The molecules were relaxed to get the optimized ground state geometry using the Generalized Gradient Approximation (GGA) of the exchange and correlation functional and Perdew-Burke-Ernzerhof (PBE) parameterization. The geometry was relaxed to a force of 0.01 eV. The periodic wave function has an energy cutoff of 250 Ry. The transmission coefficient T(E) as implemented in the GOLLUM transport code[9] for electrons of energy E (passing from the source to the drain) is expressed as follows:

$$T(E) = Tr(\Gamma_L\ (E) G^R(E) \Gamma_R\ (E) G^{R\dagger}(E)) \qquad (1)$$

where $\Gamma_{L,R}(E) = i(\sum_{L,R}(E) - {\sum_{L,R}}^{\dagger}(E))$ describes the level broadening due to the coupling between left L and right R electrodes and the central scattering region, $\Gamma_{L,R}(E)$ are the retarded self-energies associated with this coupling and $G^R = \left(ES - H - \sum_L\ - \sum_R\right)^{-1}$ is the retarded Green's function, where H is the Hamiltonian and S is the overlap matrix obtained from SIESTA implementation of DFT. The electrical conductance of the molecules were calculated from Landauer formula: $G = G_0 \int_{-\infty}^{\infty} T(E)(-\partial f/\partial E)\partial E$ where $f(E) = (e^{(E-E_F)/k_B T} + 1)^{-1}$ is the Fermi-Dirac probability distribution function, T is the temperature, E_F is the Fermi energy, $G_0 = 2e^2/h$ is the conductance quantum, e is the electron charge and h is the Planck's constant.

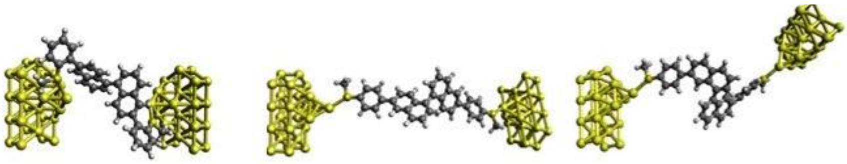

Figure S28: Structures of **1** assembled as a molecular junction.

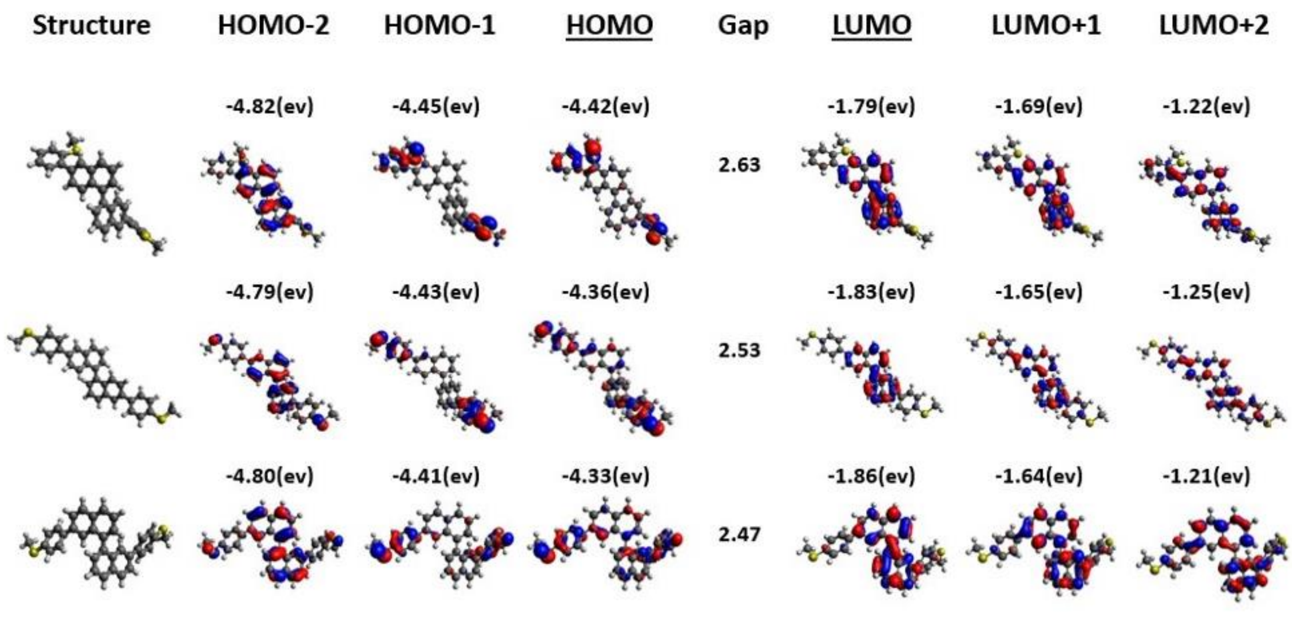

Figure S29: Molecular orbitals as a function of angle between naphthyl units of **1**

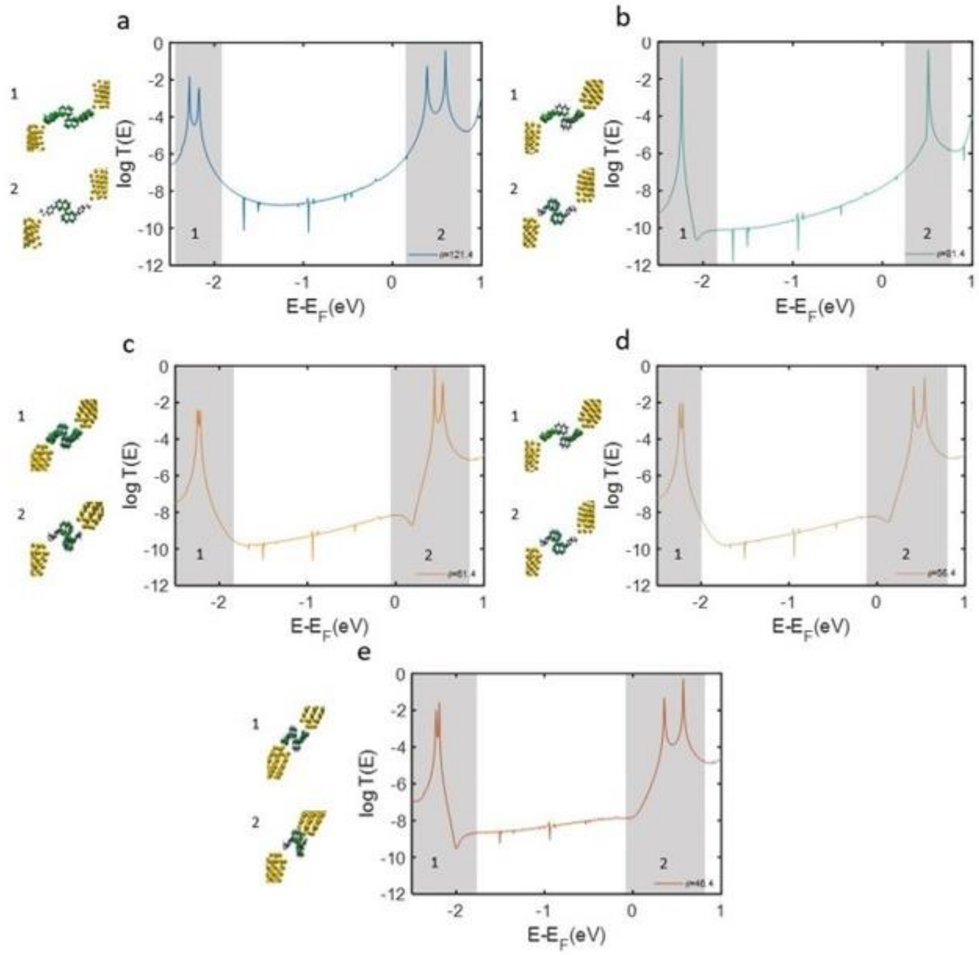

Figure S30: Local density of state calculations for the resonances due to the frontier orbitals of **1** with different angles (a-e).

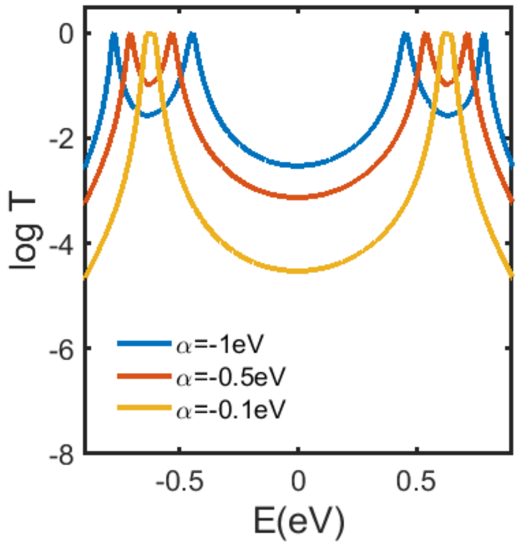

Figure S31: TB model. Transmission coefficient as a function of the electronic coupling (α) between two naphtyls in **1**.

In addition to the calculations presented in the main text, we report here the DFT-calculated transmission coefficient for compounds **2** and **3.**

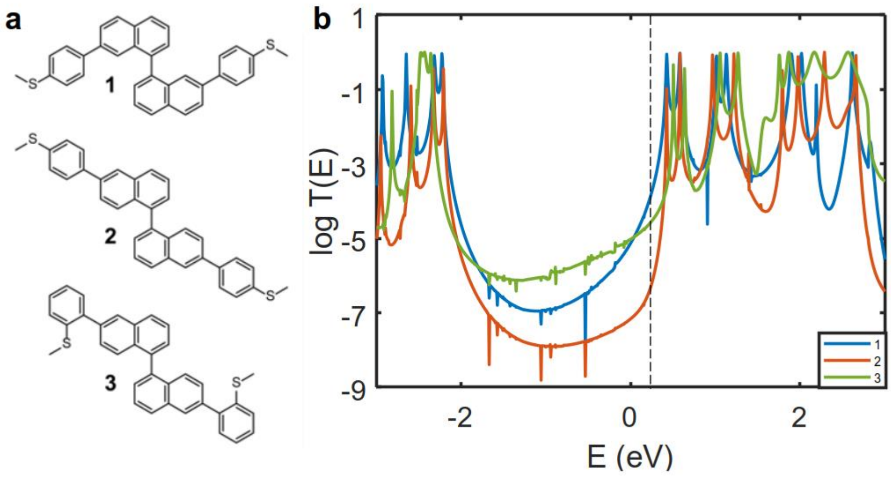

Figure S32: (a) Structures of compounds **1**, **2**, and **3** and DFT transmission coefficient of the compounds between two gold electrodes.

5.1 Wavefunction analysis upon junction compression

To clarify the origin of the quantum interference phenomena, we analysed the wavefunction on **1** in the relaxed (*transoid,* ground state) structure and in the compressed (*cisoid*) configuration.

As discussed in the main text, this is important as the transmission coefficient $T_{ij}(E)$between site i and j is proportional to the Green's function g_{ij} of a molecule, defined as

$$g_{ij}(E) = \frac{\psi_i^H \psi_j^H}{E-E_H} + \frac{\psi_i^L \psi_j^L}{E-E_L}.$$

where ψ_b^a represents the wavefunction at site b for state a, E_a is the energy level associate with this state and $a = H, L$ denote HOMO and LUMO, respectively. g_{ij} will vanish at a certain energy if the sign of product $\psi_i^H \psi_j^H$ is the same as $\psi_i^L \psi_j^L$.

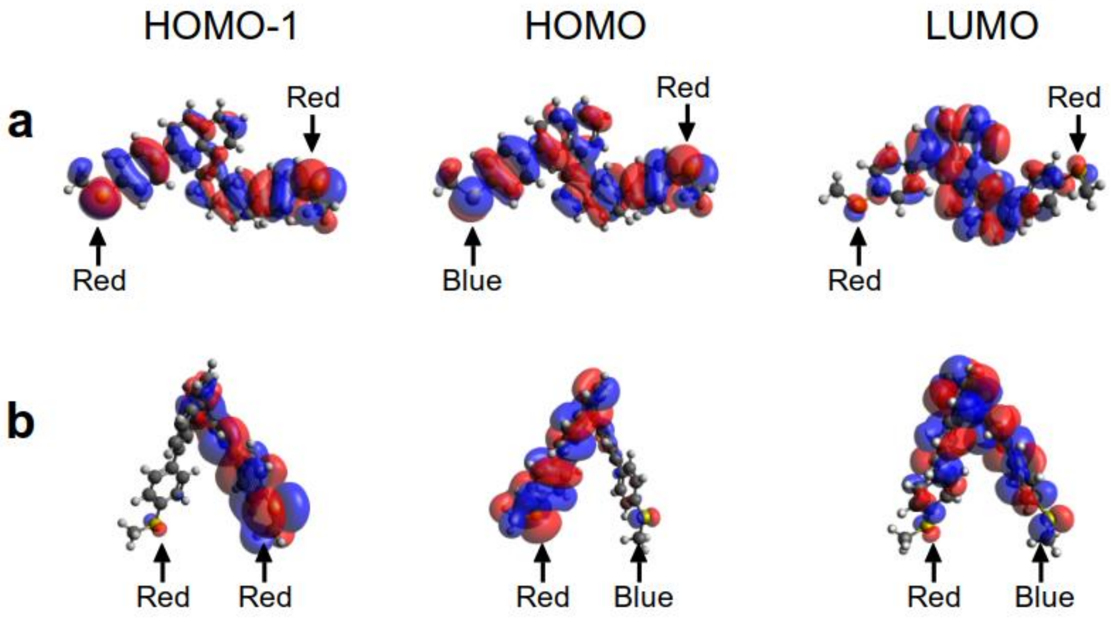

Figure S33: Molecular orbitals of molecule 1 in the *transoid* (a) and *cisoid* (b) configurations. Red and blue colours denote positive and negative wavefunction amplitude. While the sign of product of the orbital signs at the connection points to electrodes (shown by black arrows) are the same for HOMO, it changes for LUMO as a function of mechanical compression of the molecule.

As can be observed in Figure S33, the sign of the wavefunction at the electrode anchor point (the S atoms) switches in the LUMO upon junction compression, generating the QI feature discussed in the main text.

References

(1) Sbargoud, K.; Mamada, M.; Marrot, J.; Tokito, S.; Yassar, A.; Frigoli, M. Diindeno[1,2-b:2′,1′-n]Perylene: A Closed Shell Related Chichibabin's Hydrocarbon, the Synthesis, Molecular Packing, Electronic and Charge Transport Properties. *Chem. Sci.* **2015**, *6* (6), 3402–3409. https://doi.org/10.1039/C5SC00652J.

(2) Daaoub, A.; Morris, J. M. F.; Béland, V. A.; Demay-Drouhard, P.; Hussein, A.; Higgins, S. J.; Sadeghi, H.; Nichols, R. J.; Vezzoli, A.; Baumgartner, T.; Sangtarash, S. Not So Innocent After All: Interfacial Chemistry Determines Charge-Transport Efficiency in Single-Molecule Junctions. *Angewandte Chemie International Edition 62* (24), e202302150. https://doi.org/10.1002/anie.202302150.

(3) Wu, C.; Qiao, X.; Robertson, C. M.; Higgins, S. J.; Cai, C.; Nichols, R. J.; Vezzoli, A. A Chemically Soldered Polyoxometalate Single-Molecule Transistor. *Angewandte Chemie International Edition* **2020**, *59* (29), 12029–12034. https://doi.org/10.1002/anie.202002174.

(4) Naghibi, S.; Sangtarash, S.; Kumar, V. J.; Wu, J.-Z.; Judd, M. M.; Qiao, X.; Gorenskaia, E.; Higgins, S. J.; Cox, N.; Nichols, R. J.; Sadeghi, H.; Low, P. J.; Vezzoli, A. Redox-Addressable Single-Molecule Junctions Incorporating a Persistent Organic Radical. *Angewandte Chemie International Edition* **2022**, *61* (23), e202116985. https://doi.org/10.1002/anie.202116985.

(5) Wu, C.; Bates, D.; Sangtarash, S.; Ferri, N.; Thomas, A.; Higgins, S. J.; Robertson, C. M.; Nichols, R. J.; Sadeghi, H.; Vezzoli, A. Folding a Single-Molecule Junction. *Nano Letters* **2020**, *20* (11), 7980–7986. https://doi.org/10.1021/acs.nanolett.0c02815.

(6) Makk, P.; Tomaszewski, D.; Martinek, J.; Balogh, Z.; Csonka, S.; Wawrzyniak, M.; Frei, M.; Venkataraman, L.; Halbritter, A. Correlation Analysis of Atomic and Single-Molecule Junction Conductance. *ACS Nano* **2012**, *6* (4), 3411–3423. https://doi.org/10.1021/nn300440f.

(7) Soler, J. M.; Artacho, E.; Gale, J. D.; García, A.; Junquera, J.; Ordejón, P.; Sánchez-Portal, D. The SIESTA Method for Ab Initio Order- N Materials Simulation. *Journal of Physics: Condensed Matter* **2002**, *14* (11), 2745–2779. https://doi.org/10.1088/0953-8984/14/11/302.

(8) Artacho, E.; Anglada, E.; Diéguez, O.; Gale, J. D.; García, A.; Junquera, J.; Martin, R. M.; Ordejón, P.; Pruneda, J. M.; Sánchez-Portal, D.; Soler, J. M. The SIESTA Method; Developments and Applicability. *Journal of physics. Condensed matter : an Institute of Physics journal* **2008**, *20* (6), 064208. https://doi.org/10.1088/0953-8984/20/6/064208.

(9) Ferrer, J.; Lambert, C. J.; García-Suárez, V. M.; Manrique, D. Z.; Visontai, D.; Oroszlany, L.; Rodríguez-Ferradás, R.; Grace, I.; Bailey, S. W. D.; Gillemot, K.; Sadeghi, H.; Algharagholy, L. A. GOLLUM: A next-Generation Simulation Tool for Electron, Thermal and Spin Transport. *New Journal of Physics* **2014**, *16* (9), 093029. https://doi.org/10.1088/1367-2630/16/9/093029.
